# Microstructural Characterization of Short Association Fibers Related to Long‐Range White Matter Tracts in Normative Development

**DOI:** 10.1002/hbm.70255

**Published:** 2025-06-09

**Authors:** Chloe Cho, Maxime Chamberland, Francois Rheault, Daniel Moyer, Bennett A. Landman, Kurt G. Schilling

**Affiliations:** ^1^ Department of Biomedical Engineering Vanderbilt University Nashville Tennessee USA; ^2^ Department of Mathematics and Computer Science Eindhoven University of Technology Eindhoven the Netherlands; ^3^ Medical Imaging and Neuroinformatic (MINi) lab, Department of Computer Science University of Sherbrooke Canada; ^4^ Department of Computer Science Vanderbilt University Nashville Tennessee USA; ^5^ Department of Radiology and Radiological Sciences Vanderbilt University Nashville Tennessee USA; ^6^ Department of Electrical and Computer Engineering Vanderbilt University Nashville Tennessee USA; ^7^ Vanderbilt University, Institute of Imaging Science Vanderbilt University Nashville Tennessee USA

**Keywords:** diffusion MRI, microstructure, neuro‐development, superficial white matter, tractography

## Abstract

Short association fibers (SAFs) in the superficial white matter play a key role in mediating local cortical connections but have not been well‐studied as innovations in whole‐brain diffusion tractography have only recently been developed to study superficial white matter. Characterizing SAFs and their relationship to long‐range white matter tracts is crucial to advance our understanding of neurodevelopment during the period from childhood to young adulthood. This study aims to (1) map SAFs in relation to long‐range white matter tracts, (2) characterize typical neurodevelopmental changes across these white matter pathways, and (3) investigate the relationship between microstructural changes in SAFs and long‐range white matter tracts. Leveraging a cohort of 616 participants ranging in age from 5.6 to 21.9 years old, we performed quantitative diffusion tractography and advanced diffusion modeling with diffusion tensor imaging (DTI) and neurite orientation dispersion and density imaging (NODDI). Robust linear regression models were applied to analyze microstructural features, including fractional anisotropy (FA), mean diffusivity (MD), axial diffusivity (AD), radial diffusivity (RD), intracellular volume fraction (ICVF), isotropic volume fraction (ISOVF), and orientation dispersion index (ODI). Our results reveal that both SAFs and long‐range tracts exhibit similar overall developmental patterns, characterized by negative associations of MD, AD, and RD with age and positive associations of FA, ICVF, ISOVF, and ODI with age. Notably, FA, AD, and ODI exhibit significant differences between SAFs and long‐range tracts, suggesting distinct neurodevelopmental trajectories between superficial and deep white matter. In addition, significant differences were found in MD, RD, and ICVF between males and females, highlighting variations in neurodevelopment. This normative study provides insights into typical microstructural changes of SAFs and long‐range white matter tracts during development, laying a foundation for future research to investigate atypical development and dysfunction in disease pathology.

## Graphical abstract

This study investigates superficial white matter using advanced quantitative diffusion tractography. This paper addresses three key objectives: (1) mapping short association fibers in relation to long‐range white matter tracts, (2) characterizing typical neurodevelopmental changes, and (3) investigating the relationship between microstructural changes in SAFs and long‐range white matter tracts.

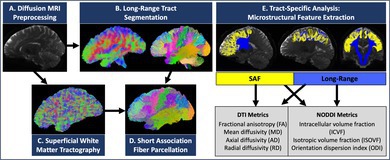

## 1 Introduction

Innovations in whole‐brain diffusion magnetic resonance imaging (MRI) tractography have allowed for in vivo reconstruction of white matter tracts, providing insights into the structural connectivity of neural pathways (Tournier 2019; Wasserthal et al. 2018; Zhang et al. 2022). While prior studies have mainly focused on long‐range tracts, the superficial white matter has been underexplored as methodological advancements have only recently been developed to track and assess superficial white matter (Chamberland et al. 2023; Schilling, Archer, Rheault, et al. 2023; Schilling, Archer, Yeh, et al. 2023; Shastin et al. 2022).

As summarized by Kirilina et al. (2020), short association fibers (SAFs) in the superficial white matter (SWM) are located directly underneath the cerebral cortex, comprising up to 60% of total white matter volume in the brain, and play a key role in mediating local cortical connections (Schüz et al. 2002; Wu et al. 2014). In healthy development, SWM and cortical thickness have been found to co‐evolve (Schilling, Archer, Rheault, et al. 2023). A recent lifespan study reported that regions with greater cortical thinning from youth to adulthood also showed greater superficial white matter expansion and thickening, suggesting coordinated maturation of the cortex and underlying U‐fiber connections (Schilling, Archer, Rheault, et al. 2023). FA has been found to linearly increase while MD and AD decrease in SWM during development before plateauing and then declining in aging (Schilling, Archer, Rheault, et al. 2023; Schilling, Archer, Yeh, et al. 2023; Van Dyken et al. 2024). SAFs, otherwise referred to as U‐fibers, are also among the final regions to myelinate, with maturation extending into the third or fourth decade of life (Kirilina et al. 2020; Schüz et al. 2002; Van Dyken et al. 2024; Wu et al. 2014). In contrast, long‐range white matter pathways generally mature earlier in life. Specifically, major commissural, projection, and association bundles begin myelinating soon after birth and often complete by early adulthood (Schilling, Chad, Chamberland, et al. 2023). The connection between SAFs and related long‐range pathways is unclear and there is currently no definitive classification of SAFs, although various approaches have been proposed, including region of interest placement, fiber clustering, and hybrid methods (Guevara et al. 2020). Prior studies on superficial white matter have found SAFs are involved in brain development, neuroplasticity, processing and integration, and dysfunction in disease (Phillips et al. 2016; Schilling, Archer, Yeh, et al. 2023; Wang et al. 2022; Wu et al. 2014). Thus, characterizing SAFs and their relationship to long‐range white matter tracts is crucial to better understand the neurodevelopmental processes that occur during the period from childhood to young adulthood.

Advanced diffusion models, including diffusion tensor imaging (DTI) and neurite orientation dispersion and density imaging (NODDI), leverage the diffusion of water molecules to infer fiber orientations and gain insights into the microstructural properties of neural pathways (Alexander et al. 2007; Tamnes et al. 2018; Zhang et al. 2012). DTI metrics include fractional anisotropy (FA), mean diffusivity (MD), axial diffusivity (AD), and radial diffusivity (RD) (Alexander et al. 2007). DTI utilizes diffusion properties to assess the directionality, organization, and integrity of white matter fibers, reflective of myelination and axonal integrity (Goddings et al. 2021; Winklewski et al. 2018). NODDI provides insights into intracellular volume fraction (ICVF), isotropic volume fraction (ISOVF), and orientation dispersion index (ODI) (Zhang et al. 2012). NODDI provides more detailed information about the microstructural environment, including the volume fractions occupied by neurites (axons and dendrites) and extracellular fluid as well as fiber orientations (Vaher et al. 2022; Zhang et al. 2012). Both models are sensitive to tissue microstructure and have been widely used to study white matter changes across development, aging, and disease (Alexander et al. 2007; Chang et al. 2015; Krogsrud et al. 2016; Schilling, Archer, Yeh, et al. 2023).

This study unites DTI and NODDI to comprehensively characterize the microstructure of SAFs in relation to long‐range tracts during neurodevelopment. Leveraging high‐resolution MRI data from a large‐scale developmental cohort, we applied robust linear regression and correlational analyses to gain insights into typical changes in neuronal structure, organization, and integrity during the important period from childhood to young adulthood as well as differences in neurodevelopmental processes between males and females. To our knowledge, this is the first study to characterize microstructural changes in corresponding SAF and long‐range bundles.

## 2 Methods

### 2.1 Data

**FIGURE 1:**
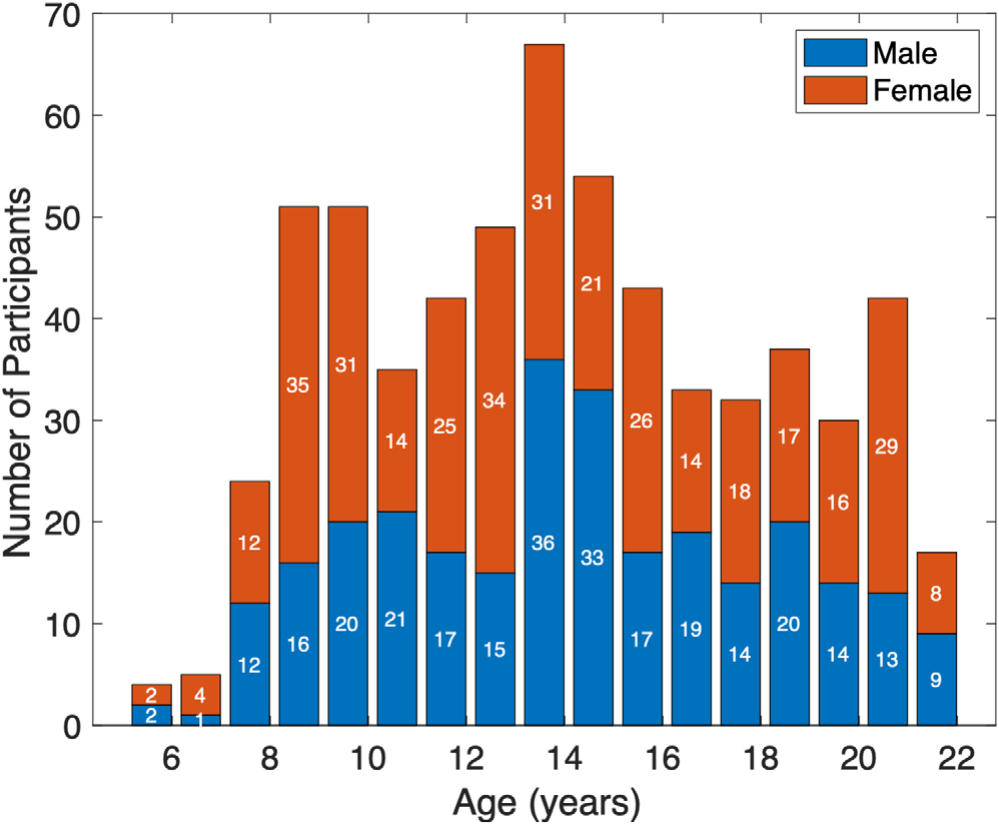
Age and sex distribution in the study cohort of 616 participants (279 M, 337 F), ranging from 5.6 to 21.9 years old with a mean age of 14.5 years.

In this study, we analyzed data from the Human Connectome Project Development (HCP‐D) database which includes MRI data to study brain connectivity and organization (Somerville et al. 2018). The HCP‐D database includes 652 participants ranging in age from 5.5 to 21.9 years. Manual quality assurance was performed on all datasets, and 36 participants from HCP‐D were excluded. Exclusion criteria included missing diffusion or T1 MRI data, failure of data processing pipelines, or excessive motion/artifacts. Our study included 616 participants (279 males, 337 females), ranging from 5.6 to 21.9 years old with a mean age of 14.5 years, who underwent structural T1 and diffusion MRI and passed quality assurance checks for MRI and tractography processes. The age and sex distribution for our study cohort are summarized in Figure 1.

Diffusion MRI was acquired using a multishell diffusion scheme, with *b*‐values of 1500 and 3000 s/mm^2^, sampled with 93 and 92 directions respectively, along with 28 b = 0 s/mm^2^ images (Somerville et al. 2018). The in‐plane resolution was 1.5 mm, and the slice thickness was 1.5 mm. Diffusion MRI preprocessing included susceptibility, motion, and eddy current corrections performed using TOPUP and EDDY algorithms from the FSL package following the minimally preprocessed HCP pipeline (Glasser et al. 2013). Structural T1‐weighted MRI was acquired using an MPRAGE sequence with a resolution of 0.8 mm isotropic.

### 2.2 Long‐Range Tract Segmentation

**FIGURE 2:**
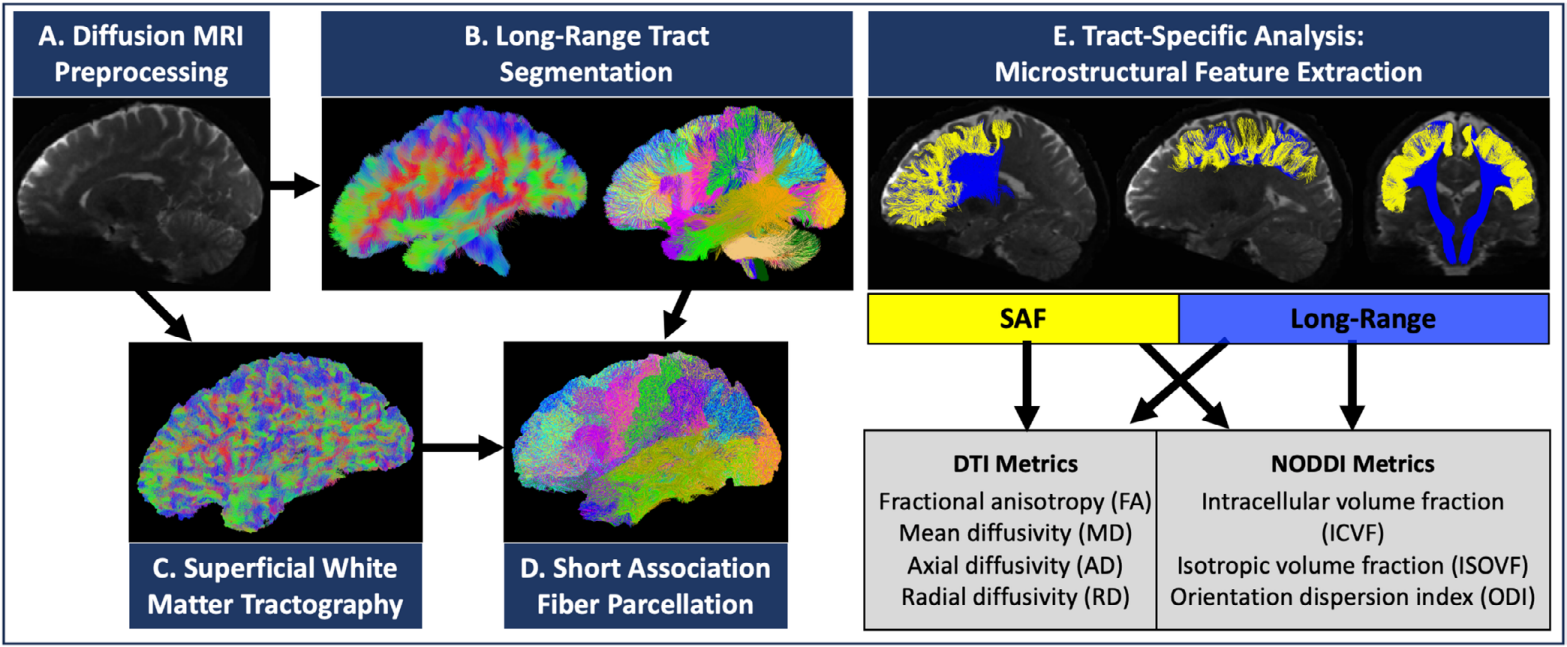
Overview of the methodological pipeline. For each participant's preprocessed diffusion MRI, automated long‐range tract segmentation, superficial white matter tractography, and parcellation of SAFs mapped in relation to long‐range bundles were conducted. DTI and NODDI metrics were extracted from each SAF and long‐range bundle to investigate the relationship between microstructural properties of SAFs and long‐range tracts.

Long‐range white matter tracts were extracted from each participant's diffusion MRI using TractSeg (Wasserthal et al. 2018), a convolutional neural network‐based algorithm to directly segment bundles based on fiber orientation distribution function (fODF) peaks. Sixty‐three white matter bundles from the TractSeg outputs were selected for analysis and classified into five (association, commissural, projection, striatal, or thalamic) major structural neural pathway groups (see Figure 2). We included the tracts where both long‐range and associated superficial white matter pathways were present in at least 95% of participants. A complete list of the bundles, classifications, and abbreviations is provided in Table S1.

### 2.3 Short Association Fiber (SAF) Tractography

The pipeline for superficial white matter tractography and SAF parcellation utilized MRtrix (Tournier et al. 2019), FSL (Jenkinson et al. 2012), and FreeSurfer (Fischl 2012) neuroimaging software. Superficial white matter tractography was performed with MRtrix, similar to the surface‐based methodology described in Shastin et al. (2022) (Shastin et al. 2022). All preprocessed diffusion MRI data were resampled to a resolution of 1 mm isotropic using the mrgrid command. dwi2fod was used to perform multi‐shell multi‐tissue constrained spherical deconvolution (Jeurissen et al. 2014) to estimate fiber orientation distributions. Diffusion and structural T1 MRI data were aligned using FSL epi_reg, a boundary‐based rigid registration method, and quality checked for proper alignment. FreeSurfer was applied to each participant's T1 MRI. The FreeSurfer aseg volume was transformed to diffusion space and inputted to MRtrix's five tissue type (5TT) image segmentation algorithm (Smith et al. 2012). The 5TT image was manipulated such that the cerebellar cortex, amygdala, hippocampus, and deep nuclei were set as gray matter volumes, creating the white/gray matter boundary for streamline seeding and forcing all streamlines to start and end at the neocortex. Anatomically constrained tractography (Smith et al. 2012) with the second‐order integration probabilistic algorithm (Tournier et al. 2010) (max angle = 45°, step size = 0.5 mm, fODF power = 0.25) was used to generate 2 million streamlines with a maximum length of 40 mm and a minimum length of 5 mm, in accordance with the definition of “short association fibers” (Schüz et al. 2002). This method for superficial white matter tractography has been previously validated, resulting in a dense system of fibers directly below the cortical sheet (Schilling, Archer, Rheault, et al. 2023; Schilling, Archer, Yeh, et al. 2023; Shastin et al. 2022).

SAFs were grouped by proximity to map SAFs in relation to the long‐range bundles from TractSeg (see Figure 2). Specifically, a binary mask of each long‐range TractSeg bundle was defined as a region of interest (ROI). Streamlines from superficial white matter tractography that passed through this ROI were assigned to the corresponding short association fiber bundle. The bundles were filtered using scilpy tools (https://github.com/scilus/scilpy) using Quickbundles hierarchical clustering (alpha = 0.6) to remove outlier streamlines (Cote et al. 2015).

### 2.4 DTI and NODDI Metrics

We characterized the microstructure of each SAF and long‐range bundle using DTI and NODDI (see Figure 2). DTI metrics included FA, MD, AD, and RD. NODDI metrics included ICVF, ISOVF, and ODI. DTI and NODDI metrics were extracted from each SAF and long‐range bundle to investigate the relationship between microstructural properties of SAFs and long‐range tracts. To generate voxel‐wise maps of FA, MD, AD, and RD, the FSL dtifit algorithm was used to fit a diffusion tensor model at each voxel where fitting was restricted to b < =1500 s/mm^2^. To generate voxel‐wise maps of ICVF, ISOVF, and ODI, the scilpy toolbox (https://github.com/scilus/scilpy) was used to fit the NODDI model (Zhang et al. 2012) at each voxel on both *b*‐value shells. For every participant, the voxel‐wise values of each feature were averaged across all voxels in a SAF or long‐range bundle to calculate the microstructural feature value associated with every bundle.

### 2.5 Analysis

#### 2.5.1 Robust Linear Regression

Robust linear regression models with the bisquare weight function were applied to analyze each feature for all long‐range and SAF bundles. We assessed the association of each feature with age, sex, age‐sex interaction term, and total intracranial volume (TICV) as described in Equation (1).

(1) $$\text{Feature}=\beta_{0}+\beta_{1}*\mathrm{Age}+\beta_{2}*\mathrm{Sex}+\beta_{3}*\mathrm{Age}*\mathrm{Sex}+\beta_{4}*\text{TICV}$$

TICV was extracted from FreeSurfer applied to each participant's structural T1 MRI. All raw feature data along with TICV were z‐transformed to ensure comparability and interpretation of model results; as a result, the beta coefficients in Equation (1) represent the association, or standard deviation change, of the feature with age, sex, age‐sex interaction term, or TICV. The Benjamini–Hochberg false discovery rate (FDR) correction was applied to control for multiple comparisons. An alpha level of 0.05 was used to determine statistical significance.

We conducted an additional regression analysis modeling TICV as a function of age, sex, and age‐sex interaction term. TICV was z‐transformed to ensure comparability and interpretation of model results, and the beta coefficients thus represent the association, or standard deviation (SD) change, in TICV. Results showed a small but statistically significant association between age and TICV (*β* = −0.04, *p* < 0.01), a nonsignificant association with sex (*β* = 0.09, *p* = 0.70), and a significant association with age‐sex interaction term (*β* = 0.07, *p* < 0.01). These results indicate diverging trajectories: TICV decreased slightly with age in females (slope = −0.04 SD/year) but increased slightly with age in males (slope = 0.03 SD/year). The observed sex‐specific trajectories for TICV are consistent with findings from prior studies that males exhibit prolonged brain growth later in adolescence compared to females who tend to reach peak brain volume earlier (Lenroot et al. 2007). We then assessed whether the effect of TICV on microstructural features varied across age or sex by evaluating the TICV‐age, TICV‐sex, and TICV‐age‐sex interaction terms in our primary model. These interaction terms were nonsignificant; therefore, the main effect of TICV was retained as an additive covariate to maintain model parsimony while appropriately accounting for anatomical variability and preserving interpretability of key predictors.

In addition, for each feature in each bundle, the relative percent change per year was calculated using the raw feature data. The slope represented the change in feature per year, accounting for the effects of sex, age‐sex interaction term, and TICV. Here, sex was coded as 0 for female and 1 for males, such that $\beta_{1}$ represents the change per year in the female model. This slope was normalized by the average feature value across all participants and multiplied by 100 to calculate the cross‐sectional relative percent change in the feature per year. The robust linear regression and 95% confidence intervals with all datapoints are shown in Figure 9 and Figure 10 for all features in five representative SAF and long‐range bundles, respectively.

#### 2.5.2 Feature Differences Between SAF and Long‐Range Pathways

To investigate differences in features between corresponding SAF and long‐range bundles, we compared the effect sizes, represented by the beta coefficients from the robust linear regression using the z‐normalized feature data. A two‐tailed t‐test was applied using the mean and standard errors for the beta coefficients. The Benjamini–Hochberg FDR correction was applied to control for multiple comparisons, and an alpha level of 0.05 was used to determine statistical significance.

#### 2.5.3 Inter‐Feature Correlational Analysis Within and Between Pathway Types

To understand the relationships among different microstructural features within and between pathway types, correlational analyses were conducted. We examined the partial correlations (controlling for age, sex, and z‐normalized TICV) between all pairs of z‐normalized feature values measured (1) within each SAF bundle (SAF‐SAF inter‐feature correlations), (2) within each long‐range bundle (LR‐LR inter‐feature correlations), and (3) between features measured in each SAF bundle and its corresponding long‐range bundle (SAF‐LR inter‐feature correlations). To calculate feature‐wise correlation coefficients and associated *p* values, we performed partial correlation analyses, controlling for age, sex, and z‐normalized TICV. The Benjamini–Hochberg FDR correction was applied to control for multiple comparisons and an alpha level of 0.05 was used to determine statistical significance. This analysis focuses on the interplay between different microstructural features, complementing the subsequent examination (Section 2.5.4) of the specificity of correlations for individual features between associated SAF‐LR pairs.

#### 2.5.4 Analysis of SAF‐LR Correlation Specificity

Following the initial correlational analyses, we investigate whether the observed relationship between a given long‐range pathway (LRᵢ) and its structurally associated SAF (SAFᵢ) was uniquely strong compared to other relationships involving LRᵢ. To quantify this specificity, we performed two sets of statistical comparisons for each microstructural feature (FA, MD, AD, RD, ICVF, ISOVF, ODI) across all 63 pathways. We specifically tested the following hypotheses:

- Hypothesis 1 (Specificity Relative to Other SAFs): Is the correlation between LRᵢ and its associated SAFᵢ significantly stronger than the average correlation between LRᵢ and other nonassociated SAFs (SAF_j_, where j ≠ i)?
- Hypothesis 2 (Specificity Relative to Other LRs): Is the correlation between LR_i_ and its associated SAF_i_ significantly stronger than the average correlation between LR_i_ and other nonassociated long‐range pathways (LR_k_, where k ≠ i)?

For each LR pathway (LRᵢ) and each feature, we utilized the partial Pearson correlation coefficients calculated in Section 2.5.3 (controlling for age, sex, and z‐normalized TICV). Specifically, for each LRᵢ, we considered: the correlation between that LR pathway and its associated SAF pathway (henceforth, the “associated SAF‐LR correlation”), the set of correlations between that LR pathway and all other nonassociated SAF pathways (the “other SAF correlations”), and the set of correlations between that LR pathway and all other nonassociated LR pathways (the “other LR correlations”).

All correlation coefficients were transformed using Fisher's z‐transformation (z = atanh(r)) to stabilize variance. To test Hypothesis 1, the z‐transformed associated SAF‐LR correlation was compared to the mean of the z‐transformed “other SAF correlations.” Similarly, for Hypothesis 2, the z‐transformed associated SAF‐LR correlation was compared to the mean of the z‐transformed “other LR correlations.” These comparisons were implemented using a one‐tailed Z‐test approximation. The Z‐score was calculated as the difference between the z‐transformed associated SAF‐LR correlation and the respective mean of the comparison set (i.e., mean of z‐transformed “other SAF correlations” or “other LR correlations”), divided by the standard error expected for a single z‐transformed partial correlation (SE = 1/sqrt(N—k—3), where N is the number of subjects and k = 3 is the number of covariates). While this approach approximates the comparison and acknowledges the statistical dependence between correlations sharing a common variable (LRᵢ), it provides a quantitative estimate of specificity.

One‐tailed *p* values were derived from the resulting Z‐scores for each hypothesis, pathway, and feature. To account for multiple comparisons across the 63 pathways, Benjamini–Hochberg FDR correction was applied to control for multiple comparisons, and an alpha level of 0.05 was used to determine statistical significance. Significance for Hypothesis 1 was interpreted as evidence that the *LRᵢ‐SAFᵢ relationship is specific relative to other SAF systems*, indicating the designated SAF partner stands out compared to how LRᵢ relates, on average, to the superficial fiber systems. Significance for Hypothesis 2 was interpreted as evidence for *specificity relative to other LR systems*, indicating the LRᵢ‐SAFᵢ link stands out compared to relationships between LRᵢ and other major long‐range pathways. Evaluating both hypotheses provides insight into the degree and nature of the specificity for each pathway‐feature pair.

## 3 Results

### 3.1 SAF Classification in Relation to Long‐Range Tracts

**FIGURE 3:**
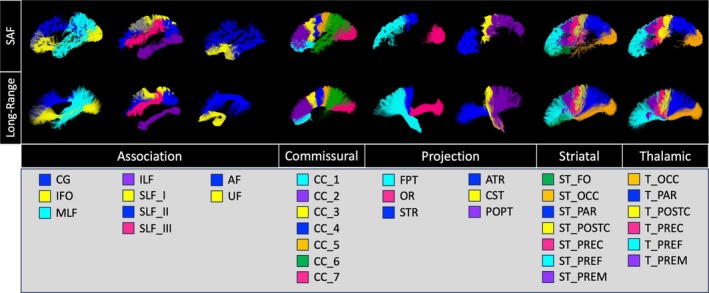
Mapping SAFs in relation to long‐range white matter tracts. Diffusion tractography and white matter segmentation methods were successfully applied across study cohort participants. The long‐range bundles were further organized into structural neural pathway groups (association, commissural, projection, striatal, or thalamic). SAFs are grouped by proximity in relation to these long‐range bundles. A complete list of bundles names and abbreviations is provided in Table S1.

Diffusion tractography and white matter segmentation methods were successfully applied across all study cohort participants to extract the corresponding SAF and long‐range bundles shown in Figure 3. The long‐range bundles were further organized into structural neural pathway groups (association, commissural, projection, striatal, or thalamic). SAFs are grouped by proximity to map SAFs in relation to these long‐range bundles. For example, the corticospinal tract (CST) is a long‐range projection pathway. However, its cortical connections have associated SAFs. Thus, the “CST SAF bundle” refers to SAFs that are structurally associated with the long‐range CST pathway. A complete list of bundles names and abbreviations is provided in Table S1.

### 3.2 How Do Microstructural Features Change Over Developmental Age in SAFs Compared to Long‐Range Tracts?

**FIGURE 4:**
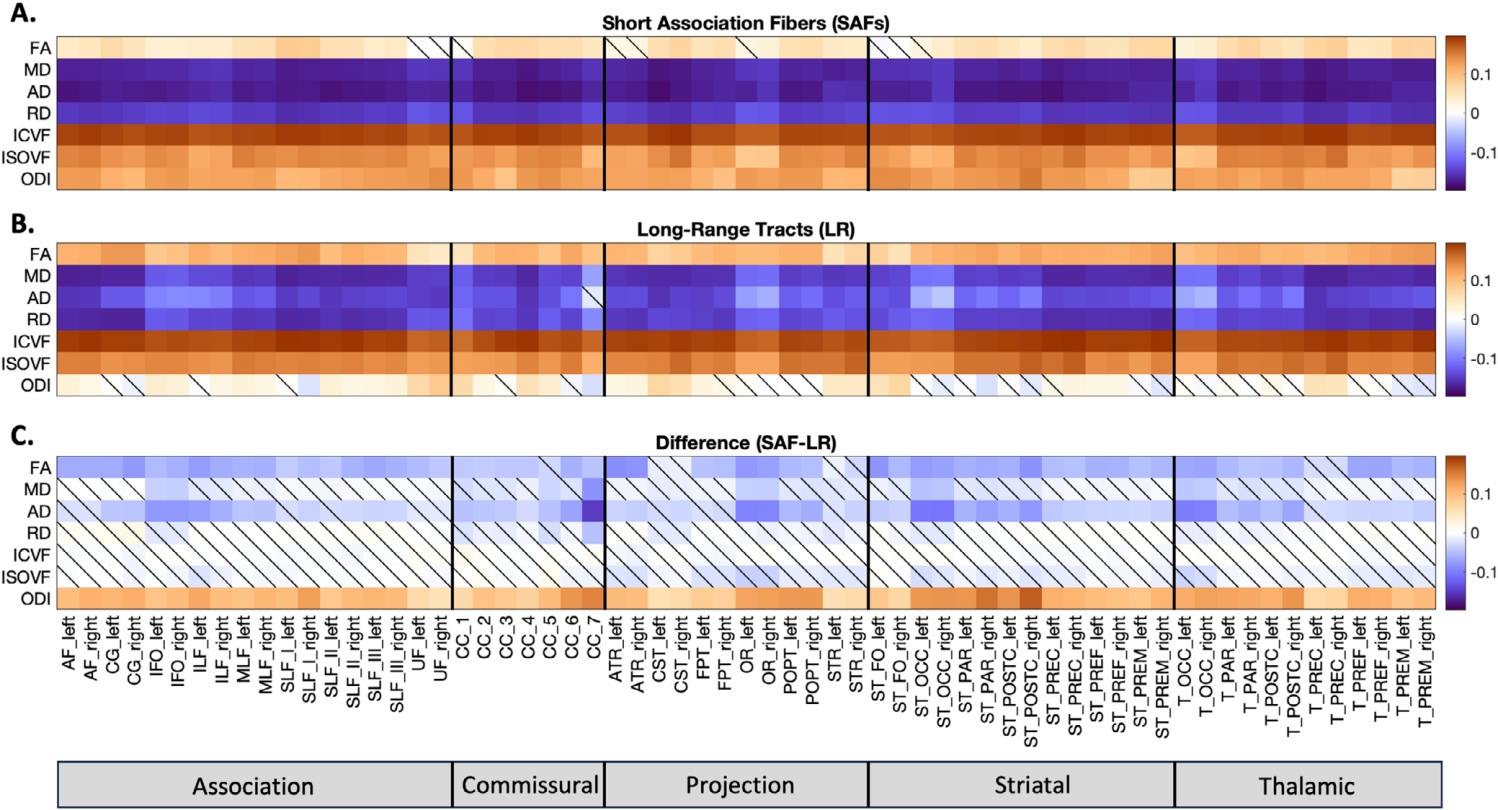
Associations of Microstructural Features with Age. (A and B) Significant associations between age and microstructural features of SAF and long‐range bundles are observed across all pathways. The $\beta_{1}$ coefficient from robust linear regression using z‐normalized feature data is plotted as a matrix for all pathways and their associated microstructural features. The colorscale represents the effect size, $\beta_{1}$, which is the association (standard deviation change) of the feature with age. Significance was determined by alpha = 0.05 after FDR correction (nonsignificant correlations are shown with a diagonal line). (C) Corresponding SAF and long‐range bundles have significant differences in effect sizes for FA, AD, and ODI. Significance (alpha = 0.05) was determined using a two‐tailed t‐test with Benjamini–Hochberg FDR correction.

Microstructural features across all SAF and long‐range bundles were found to have significant age associations over the developmental period from childhood to young adulthood as shown in Figure 4. Our results reveal that both SAFs and long‐range tracts exhibit similar overall developmental patterns, characterized by negative associations of MD, AD, and RD with age and positive associations of FA, ICVF, ISOVF, and ODI with age. Notably, SAFs exhibited smaller changes in FA and AD and larger changes in ODI compared to corresponding long‐range tracts, suggesting distinct neurodevelopmental trajectories between these pathways.

**FIGURE 5:**
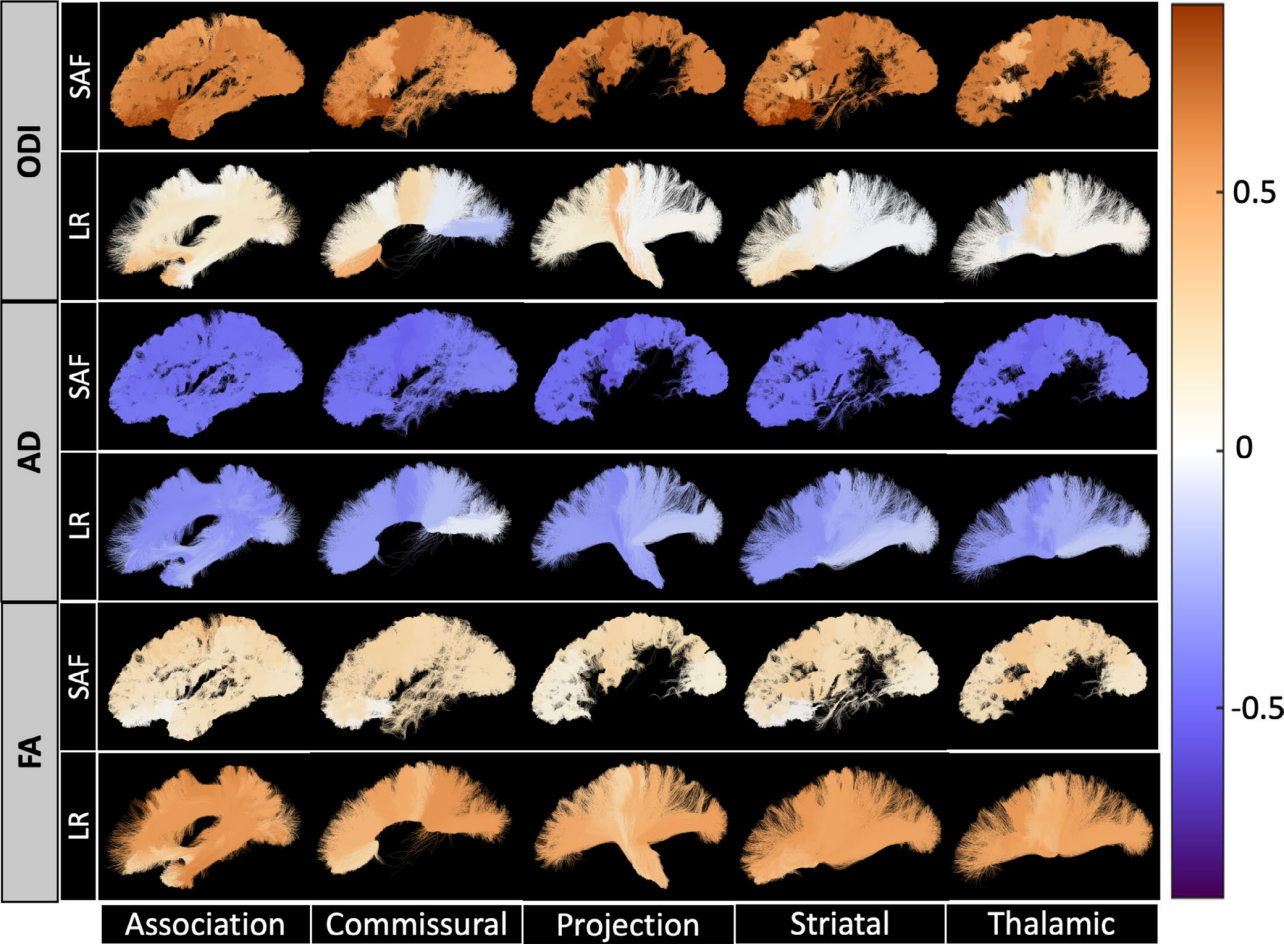
FA, AD, and ODI demonstrate spatial patterns and distinct trajectories between corresponding SAF and long‐range bundles throughout the brain. The colorscale shows the cross‐sectional relative change in the feature per year as a percent of the population mean. The data for all features and regions of interest are presented in Table S2.

In addition, we analyzed the relative change in the feature per year as a percent of the population mean. Visualizations of the relative percent changes per year in FA, AD, and ODI throughout the brain are shown in Figure 5, demonstrating spatial patterns and distinct trajectories in SAF and long‐range bundles. Specifically, during development, SAF bundles had lower rates of increase in FA (SAF range = [−0.02, 0.37] with median = 0.23 vs. LR range = [0.22, 0.68] with median = 0.51), higher rates of decrease in AD (SAF range = [−0.39, −0.55] with median = −0.46 vs. LR range = [−0.04, −0.39] with median = −0.30), and higher rates of increase in ODI (SAF range = [0.43, 0.87] with median = 0.63 vs. LR range = [−0.19, 0.53] with median = 0.10) compared to their corresponding long‐range bundles. The data for all features in each SAF and LR bundle are presented in Table S2. Furthermore, notable spatial patterns over development include anterior–posterior patterns of change in AD and FA with more heterogeneous changes in ODI. The anterior–posterior spatial patterns are more prominent in long‐range pathways with more homogeneous changes in the corresponding SAF pathways.

**FIGURE 6:**
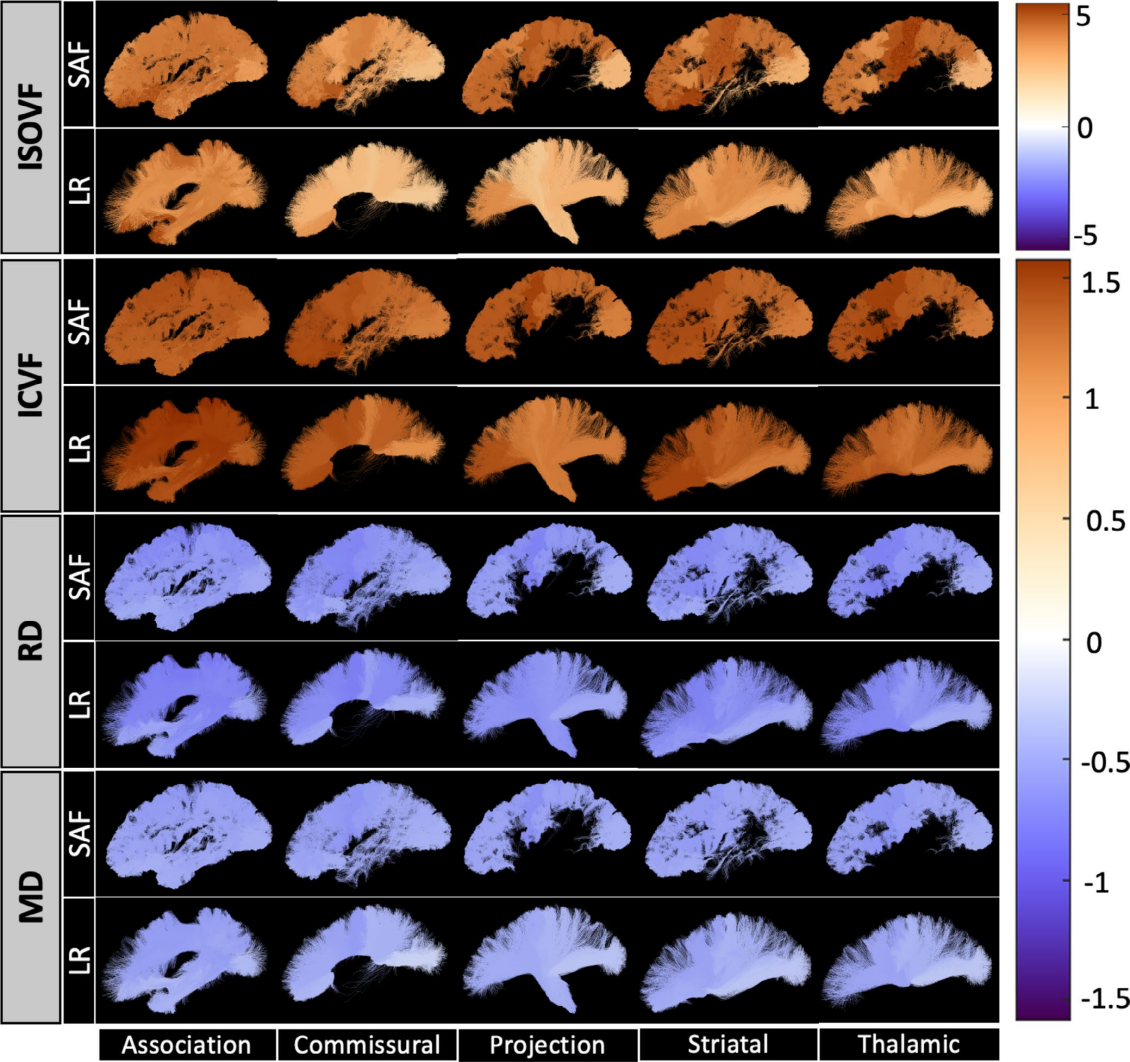
Similar changes in MD, RD, ICVF, and ISOVF over age are observed between corresponding SAF and long‐range bundles throughout the brain. The colorscale shows the cross‐sectional relative change in the feature per year as a percent of the population mean. The data for all features and regions of interest are presented in Table S2.

Visualizations of the relative percent changes per year in MD, RD, ICVF, and ISOVF throughout the brain are shown in Figure 6, demonstrating spatial patterns and similar trajectories in SAF and long‐range bundles. Spatially, anterior–posterior patterns of change are observed in ISOVF and ICVF in both SAF and long‐range bundles. The yearly relative percent changes did not significantly differ between corresponding SAF and long‐range bundles for MD (SAF range = [−0.43, −0.64] with median = −0.52 vs. LR range = [−0.22, −0.57] with median = −0.46), RD (SAF range = [−0.45, −0.74] with median = −0.59 vs. LR range = [−0.44, −0.80] with median = −0.65), ICVF (SAF range = [1.16, 1.54] with median = 1.38 vs. LR range = [1.09, 1.58] with median = 1.38), and ISOVF (SAF range = [2.42, 5.47] with median = 4.30 vs. LR range = [2.13, 5.36] with median = 3.73). The data for all features in each SAF and LR bundle are presented in Table S2.

### 3.3 Are There Differences in Microstructure Between Males and Females?

**FIGURE 7:**
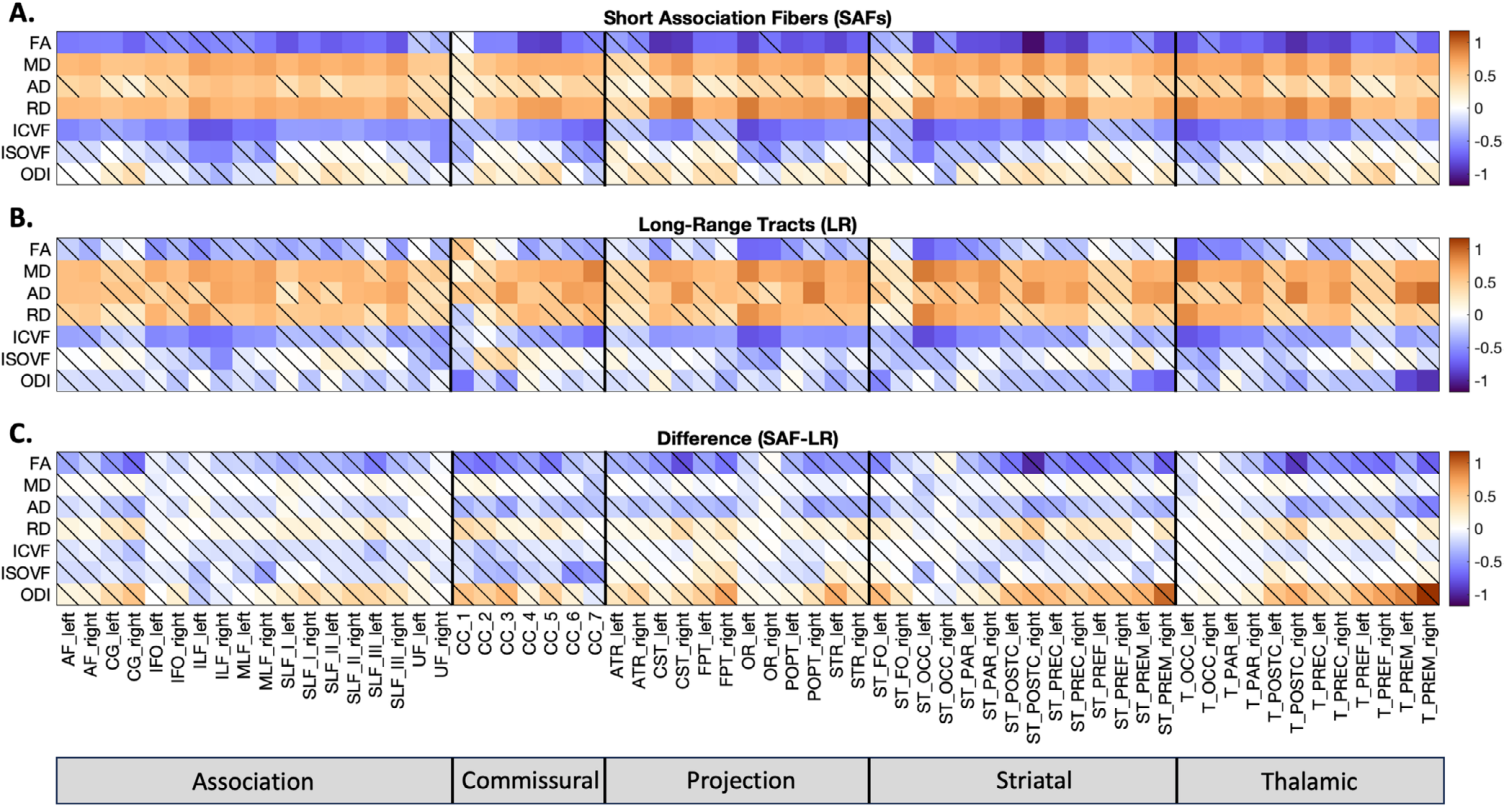
Sex Differences in Microstructural Features. (A & B) Significant differences in SAF (FA, MD, RD, and ICVF) and long‐range (MD, AD, RD, and ICVF) bundle microstructure between males and females are observed across all pathways. The $\beta_{2}$ coefficient from robust linear regression using z‐normalized feature data is plotted as a matrix for all pathways and their associated microstructural features. The colorscale represents the effect size, $\beta_{2}$, which is the association (standard deviation change) of the feature with sex (0 = female, 1 = male). Significance was determined by alpha = 0.05 after FDR correction (nonsignificant correlations are shown with a diagonal line). (C) Corresponding SAF and long‐range bundles did not have significant differences in effect sizes. Significance (alpha = 0.05) was determined using a two‐tailed t‐test with Benjamini–Hochberg FDR correction.

Notable differences in microstructural features were detected between males and females, which are summarized in Figure 7. In SAF bundles, FA and ICVF had significant negative associations with sex (0 = female, 1 = male), indicating lower baseline values for FA and ICVF in males compared to females. Conversely, MD and RD had significant positive associations, indicating higher baseline values for these features in males. Similar trends were observed in the corresponding long‐range tracts, with lower baseline values for ICVF and higher baseline values for MD, AD, and RD in males. For both SAF and long‐range bundles, there were pathway‐specific variations in the effect sizes and significance of these associations, as shown in Figure 7. Furthermore, the effect sizes across features were similar for corresponding SAF and long‐range bundles.

### 3.4 Is There an Age–Sex Interaction Effect on Neurodevelopment?

**FIGURE 8:**
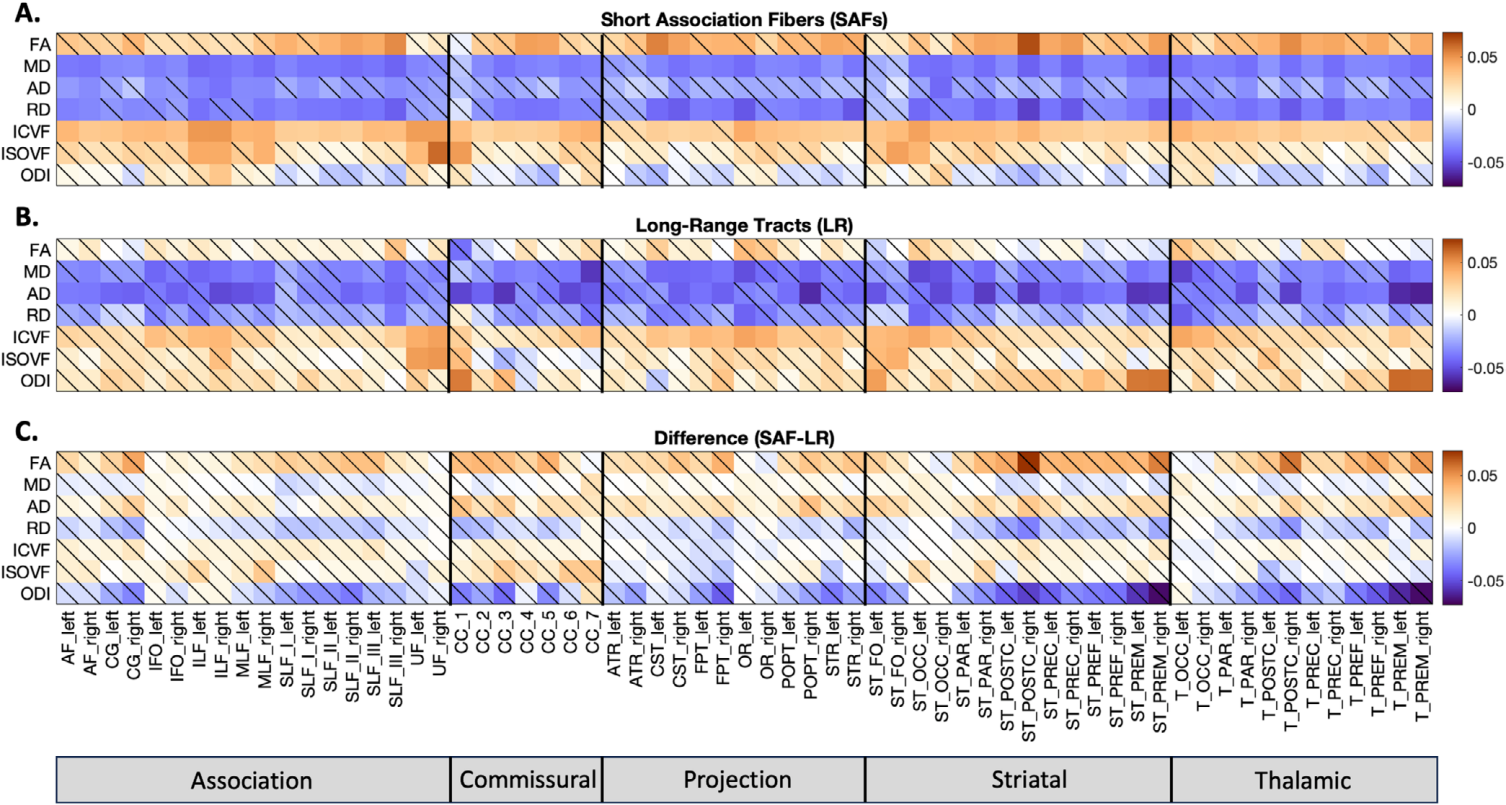
Age‐Sex Interaction Effects on Microstructural Features. (A & B) There are significant age‐sex interaction effects on SAF (MD, RD, ICVF) and long‐range bundle (MD, AD, ICVF) microstructure. The $\beta_{3}$ coefficient from robust linear regression using z‐normalized feature data is plotted as a matrix for all pathways and their associated microstructural features. The colorscale represents the effect size, $\beta_{3}$, which is the association (standard deviation change) of the feature with the age‐sex interaction term. Significance was determined by alpha = 0.05 after FDR correction (nonsignificant correlations are shown with a diagonal line). (C) Corresponding SAF and long‐range bundles did not have significant differences in effect sizes. Significance (alpha = 0.05) was determined using a two‐tailed t‐test with Benjamini–Hochberg FDR correction.

**FIGURE 9:**
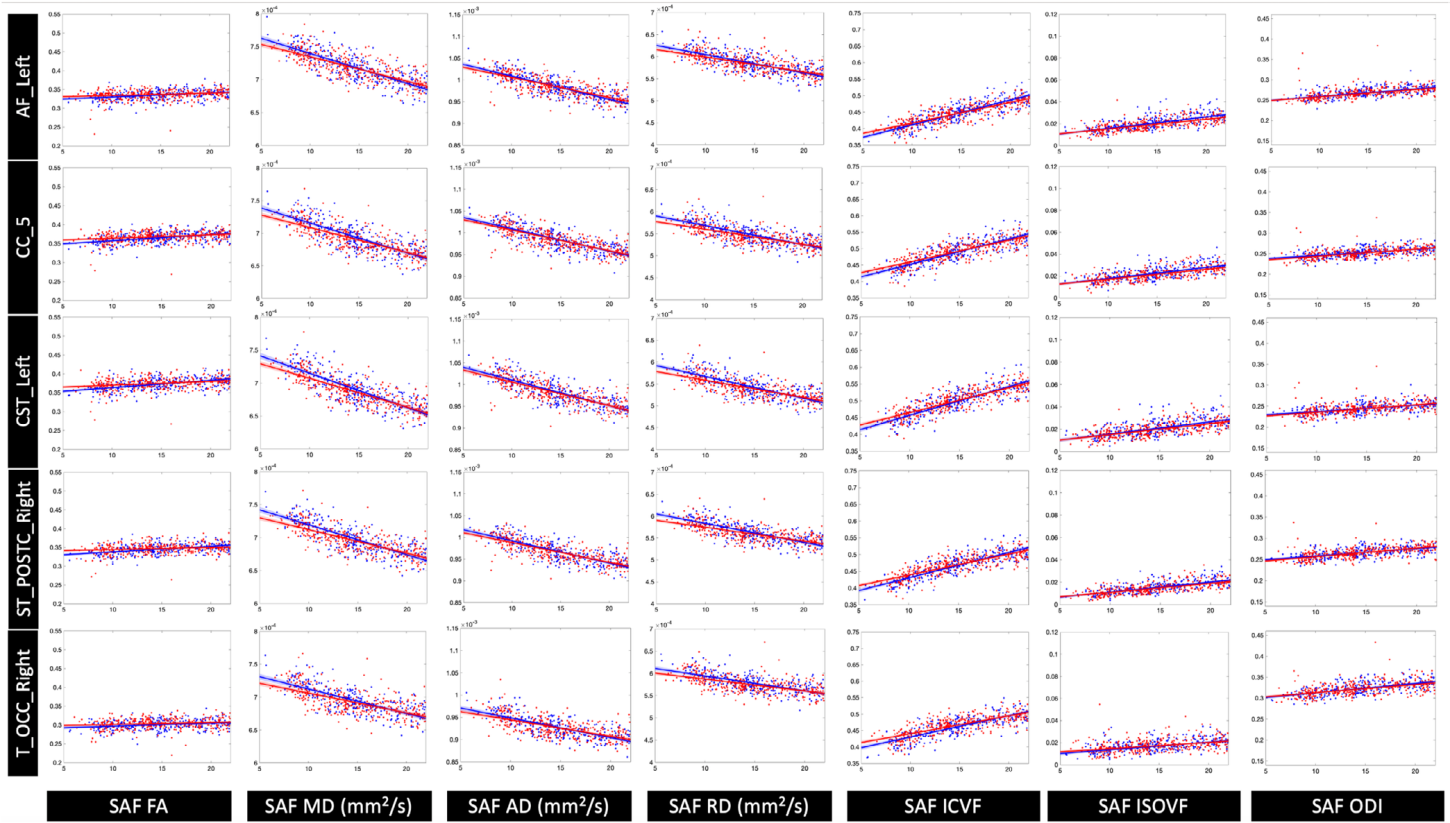
Changes in the microstructure of SAF bundles with age are observed across all pathways. Five representative pathways (one from each of the structural neural pathway groups) are shown with all participant data adjusted for differences in TICV and plotted against age in years (blue = male, red = female). Robust linear regression models with 95% confidence intervals are shown with both male and female trajectories over the developmental age range of our study cohort.

**FIGURE 10:**
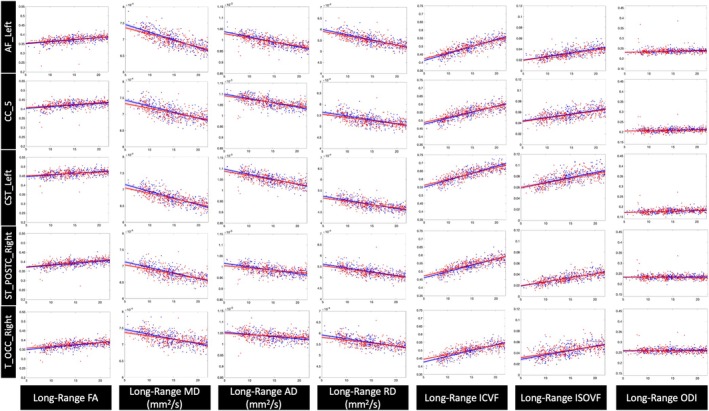
Changes in the microstructure of long‐range bundles with age are observed across all pathways. Five representative pathways (one from each of the structural neural pathway groups) are shown with all participant data adjusted for differences in TICV and plotted against age in years (blue = male, red = female). Robust linear regression models with 95% confidence intervals are shown with both male and female trajectories over the developmental age range of our study cohort.

Significant age–sex interaction effects were identified for both SAF and long‐range bundles, as summarized in Figure 8. In SAF bundles, MD and RD had significant negative associations with the age–sex interaction term, indicating higher cross‐sectional rates of decrease in MD and RD in males compared to females over this neurodevelopmental period. Similar effects for MD and AD were found in long‐range bundles. For both SAF and long‐range bundles, ICVF had significant positive associations with the age–sex interaction term, indicating higher cross‐sectional rates of increase in ICVF in males. Notably, the interaction effects exhibited pathway‐specific variations as shown in Figure 8. The male and female trajectories over development for all features in five representative SAF and long‐range bundles are shown in Figure 9 and Figure 10, respectively. The effect sizes across features were similar for corresponding SAF and long‐range bundles.

### 3.5 Are There Correlations Among SAF and Long‐Range Bundle Features?

**FIGURE 11:**
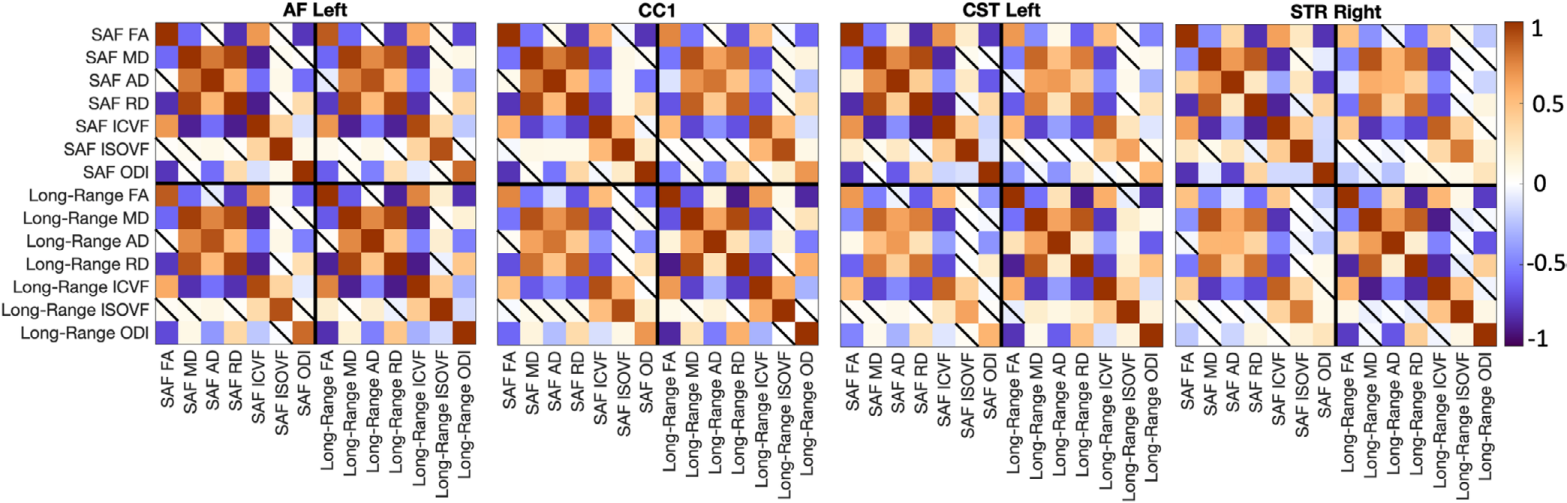
Feature‐wise correlations. SAF and long‐range bundles demonstrate similar overall correlational patterns when assessing (1) the relationship of features within each SAF bundle, (2) the relationship of features within each long‐range bundle, and (3) the relationship of SAF bundle features with their corresponding long‐range bundle features. For four representative bundles, partial correlation coefficients are shown with significance determined by alpha = 0.05 after FDR correction (nonsignificant correlations are shown with a diagonal line). Correlational matrices for all bundles are included in Figure S1.

We assessed the relationship of z‐normalized feature values for (1) each SAF bundle (SAF‐SAF), (2) each long‐range bundle (LR‐LR), and (3) each SAF bundle with its corresponding long‐range bundle (SAF‐LR) using partial correlational analyses, controlling for the effects of age, sex, and TICV. Correlation coefficients and their significance in four representative pathways are shown in Figure 11. Correlation matrices for all tracts are included in Figure S1. While there is pathway‐specific variation in the strength and significance of the correlations, the feature‐wise correlations demonstrated consistent trends across pathways.

Strong positive correlations in SAF‐SAF, LR‐LR, and SAF‐LR feature comparisons were observed between FA and ICVF; MD and RD; and MD and AD. Interestingly, MD was more strongly correlated with RD than with AD across all pathways. Strong negative correlations in both SAF‐SAF and LR‐LR comparisons were observed between FA and RD; MD and ICVF; RD and ICVF; and FA and ODI. Similarly, SAF‐LR comparisons showed strong negative correlations between FA and RD; MD and ICVF; and RD and ICVF. Notably, while FA and RD had strong negative correlations across pathways, the relationship between FA and AD was variable, suggesting that changes in RD drive changes in FA. Furthermore, SAF‐LR comparisons also showed strong positive correlations between corresponding features in the SAF and long‐range bundles, particularly for MD, RD, ICVF, and ISOVF.

### 3.6 Specificity of SAF‐LR Microstructural Correlations

**FIGURE 12:**
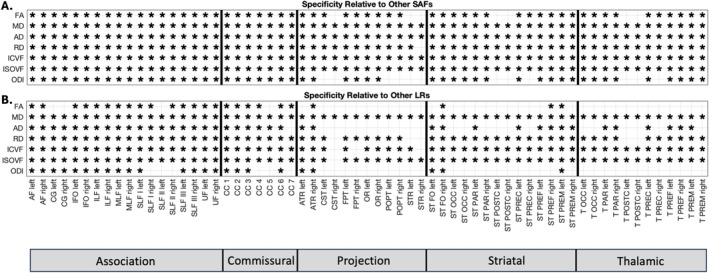
Specificity of correlations between LR pathways and associated SAFs. **(A)** Results for Hypothesis 1: Testing if the correlation between an LR pathway and its structurally linked SAF is significantly stronger than the average correlation between that LR pathway and other SAF systems. (B) Results for Hypothesis 2: Testing if the correlation between an LR pathway and its associated SAF is significantly stronger than the average correlation between that LR pathway and other LR systems. An asterisk (*) indicates hypothesis is confirmed with significance determined by alpha = 0.05 after FDR correction.

To assess the specificity of the relationship between LR pathways and their associated SAFs, we tested whether the partial correlation for each pathway‐feature pair was significantly stronger than the average correlation between the LR pathway and either other nonassociated SAFs (Hypothesis 1) or other nonassociated LRs (Hypothesis 2). The significance patterns across all features and pathways, after FDR correction, are displayed in Figure 12.

Testing Hypothesis 1 (Specificity Relative to Other SAFs) revealed that the LRᵢ‐SAFᵢ correlation was significantly stronger than the average correlation between LRᵢ and other SAF systems for most features and pathways (see Figure 12A). Widespread significance was observed across nearly all pathways for MD, AD, RD, ICVF, and ISOVF. Significance was also prevalent for FA and ODI, although some pathways, particularly projection pathways to pre/post‐central gyri (e.g., CST, STR, ST_POSTC), did not reach significance for these two features.

Testing Hypothesis 2 (Specificity Relative to Other LRs) revealed varied patterns of specificity when comparing the LRᵢ‐SAFᵢ correlation to the average correlation between LRᵢ and other LR systems (see Figure 12B). Strong significance was found across most pathways for MD, RD, ICVF, and ISOVF. AD also demonstrated significance across many pathways, although nonsignificant results were somewhat more frequent compared to other diffusivity measures. In contrast, FA and ODI showed more pathway group‐dependent results for Hypothesis 2, with statistically significant findings in association pathways while most commissural and projection pathways did not have statistically stronger relationships between LR_i_‐SAF_i_ than between LR_i_ and other LR, indicating that the correlation between an LR pathway and its associated SAF was generally not stronger than the average correlation observed between that LR pathway and other long‐range tracts.

Overall, these results indicate distinct specificity patterns depending on the microstructural feature. Diffusivity (MD, RD, AD) and volume fraction (ICVF, ISOVF) measures frequently showed significant specificity relative to both other SAF systems and other LR systems. In contrast, features reflecting anisotropy and orientation dispersion showed more limited specificity, primarily appearing significant relative to other SAF systems (Hypothesis 1) but generally not relative to other LR systems (Hypothesis 2).

## 4 Discussion

Using high‐resolution MRI data from a large‐scale developmental cohort, we applied quantitative diffusion tractography to map SAFs in relation to known long‐range white matter tracts. Uniting advanced DTI and NODDI diffusion models, we comprehensively characterized SAFs and long‐range tracts, analyzed the effects of age and sex on tissue microstructure, and examined the relationships between microstructural features in corresponding SAF and long‐range bundles.

To our knowledge, this is the first study to characterize microstructural changes in corresponding SAF and long‐range bundles. We find that microstructural changes occur over developmental age, with distinct neurodevelopmental trajectories between superficial and deep white matter. Furthermore, we identified significant sex and age‐sex interaction effects on white matter development, suggesting differing developmental trajectories between males and females. By mapping SAFs in relation to long‐range tracts, this study advances our understanding of superficial and deep white matter microstructure during the important period from childhood to young adulthood.

### 4.1 Developmental Changes in SAF vs. Long‐Range Bundle Microstructure

We found that there are continual changes in white matter microstructure over the period from childhood to young adulthood. SAF and long‐range bundles exhibited similar overall developmental trends, characterized by negative associations of MD, AD, and RD with age and positive associations of FA, ICVF, ISOVF, and ODI with age. These findings are consistent with current literature in the field showing increases in FA and neurite density and decreases in MD and RD across development in children and adolescent populations (Krogsrud et al. 2016; Mah et al. 2017; Reynolds et al. 2019; Schmithorst and Yuan 2010). The similarity in trends between SAF and long‐range bundles is not surprising as we would expect to see white matter maturation across tracts, both deep and superficial, over this developmental age range (Lebel and Beaulieu 2011; Lebel and Deoni 2018; Schilling, Chad, Chamberland, et al. 2023). We also observed tract‐specific variation in the features, particularly ODI in long‐range tracts. This reaffirms prior work studying brain development using NODDI that found that there are region‐specific maturation processes (Zhao et al. 2021).

This study also provides further insights into the neurodevelopmental and organizational differences between superficial and deep white matter. Superficial white matter is known to be among the final regions to myelinate, continuing to mature into adulthood (Kirilina et al. 2020; Schüz et al. 2002; Wu et al. 2014). We found that FA, AD, and ODI exhibited significant differences between SAFs and related long‐range tracts. Specifically, SAFs had lower rates of increase in FA, higher rates of decrease in AD, and higher rates of increase in ODI. These findings indicate that the smaller and more disperse fibers, reflected by lower FA values, in the superficial white matter appear to undergo more complex maturation and reorganizational processes with crossing fibers and less coherent axons during development, evidenced by the simultaneous increases in ICVF and ODI and decreases in AD. This discrepancy suggests that during adolescence, an individual may have relatively mature long‐range pathways but still developing local circuits. These findings are also interesting in light of recent work indicating superficial white matter and cortical thickness co‐evolve (Schilling, Archer, Rheault, et al. 2023). Because SAF myelination overlaps with periods of synaptic pruning and cortical thinning (Tamnes et al. 2013), this suggests an interplay between gray matter development and the insulation of short‐range fibers. Functionally, this may contribute to the refinement of higher‐order cognitive abilities throughout adolescence, as local networks, supported by SAFs, catch up to more established global networks made up of long‐range tracts.

Overall, changes in tissue microstructure during this developmental period are reflective of global white matter maturation, with increased neuronal density and myelination. Furthermore, complex organizational changes give rise to distinct neurodevelopmental trajectories between superficial and deep white matter, potentially due to unique structural roles, environmental differences such as vasculature and gray matter influences, and susceptibility to physiological influences (Kirilina et al. 2020).

### 4.2 Male vs. Female Neurodevelopment

Our analyses of the sex and age‐sex interaction effects on white matter development revealed significant differences in DTI and NODDI microstructural features between males and females. While these effects exhibited tract‐specific variations for both SAF and long‐range bundles, notable trends were found across regions for MD, RD, and ICVF. Specifically, MD and RD exhibited higher baseline values and higher cross‐sectional rates of decrease, while ICVF exhibited lower baseline values and higher cross‐sectional rates of increase in males compared to females.

These results complement other studies on white matter development in children and adolescents that found females undergo earlier white matter maturation and exhibit slower microstructural changes during development (Asato et al. 2010; Lebel and Deoni 2018; Reynolds et al. 2019; Schmithorst and Yuan 2010). Furthermore, our results are consistent with previously reported differences in microstructural features during childhood between males and females, such as higher MD, AD, and RD values with region‐specific differences in FA for males compared to females (Ladouceur et al. 2012; Lawrence et al. 2023; Schmithorst and Yuan 2010). Finally, our study analyzed changes over a larger developmental period from childhood to young adulthood. The differences in baseline feature values and cross‐sectional rates of change unify findings from prior studies showing lower FA/higher diffusivity values during childhood and higher FA/lower diffusivity values during adulthood in males compared to females (see Figures 9 and 10) (Asato et al. 2010; Lawrence et al. 2021; Lawrence et al. 2023; Menzler et al. 2011; Simmonds et al. 2014).

The findings from our study provide a more complete picture of differing neurodevelopmental trajectories between males and females, specifically that (1) earlier white matter maturation occurs in females, (2) steeper, more protracted microstructural changes across the developmental period from childhood to young adulthood occur in males, and (3) differences between male and female white matter development appear to be driven by increased neurite density and myelination as reflected by RD and ICVF. Interestingly, the effect sizes on corresponding SAF and long‐range bundles were similar, suggesting consistent effects of sex and age‐sex interaction across superficial and deep white matter.

### 4.3 Feature‐Wise Correlations

Feature‐wise correlational analyses, controlling for the effects of age, sex, and TICV, demonstrated consistent trends in both SAF and long‐range bundles across all pathways. In both SAF‐SAF and LR‐LR comparisons, strong positive correlations were found between FA and ICVF, MD and RD, and MD and AD. Strong negative correlations were observed between FA and RD, MD and ICVF, RD and ICVF, and FA and ODI. These feature‐wise correlations were reflective of maturation of neurites and myelination with increased structural integrity and organization during healthy development. Interestingly, MD was more strongly correlated with RD than with AD across all pathways, and FA was strongly correlated with RD. Taken together, our results suggest that, while axonal coherence and maturation occur, myelination and increased fiber density drive these microstructural changes in neurodevelopment. These findings also align with prior literature that myelination and increased fiber density enhance structural integrity and organization in neurodevelopment (Chang et al. 2015; Goddings et al. 2021; Mah et al. 2017; Winklewski et al. 2018).

Furthermore, by mapping SAFs in relation to long‐range white matter tracts, this is the first study, to our knowledge, that has examined how structurally related SAFs and LR bundles develop. Specifically, similar to the SAF‐SAF and LR‐LR comparisons, SAF‐LR comparisons showed strong positive correlations between FA and ICVF; MD and RD; and MD and AD, while strong negative correlations were found between FA and RD; MD and ICVF; and RD and ICVF. This was particularly interesting because, while long‐range tracts connect distant brain regions supporting global communication and multimodal integration, SAFs connect cortico‐cortical areas and are thought to optimize brain efficiency through dense local networks (Schilling, Archer, Rheault, et al. 2023; Schilling, Archer, Yeh, et al. 2023; Van Dyken et al. 2024). These findings suggest the relationships among microstructural features were consistent across superficial and deep white matter bundles, adding a novel dimension to our understanding of white matter maturation during development and indicating common underlying biological mechanisms affecting superficial and deep white matter maturation across the brain globally. A limitation in the feature‐wise correlations is that there are dependencies among these microstructural features that may not necessarily be driven by underlying structural changes in the brain but rather by overlap in measurement methods. It is important to note that, while the correlational feature‐wise relationships demonstrated consistent patterns, differences between superficial and deep white matter with age and sex indicate that the expression and impact of these underlying mechanisms may be affected by unique structural roles, depth‐related environmental differences, and susceptibility to physiological influences (Kirilina et al. 2020).

### 4.4 Specificity and Nature of SAF‐LR Microstructural Coupling

Our findings demonstrate feature‐dependent specificity in the coupling between LR tracts and their associated SAFs (see Figure 12). For metrics reflecting tissue composition—such as water content, axonal packing, and compartmental boundaries (MD, RD, ICVF, and ISOVF)—we observed consistently stronger correlations within LR‐SAF pairs than with unrelated SAFs or LR systems. These volume‐ and diffusion‐based metrics exhibit strong pair‐specific associations, suggesting tightly coordinated microstructural integrity within LR‐SAF units. AD showed a similar pattern, though with slightly reduced specificity across long‐range comparisons, potentially reflecting more global influences on axial coherence.

In contrast, metrics reflecting fiber geometry—FA and ODI—showed limited specificity. Both features exhibited significant coupling only relative to unrelated SAFs, with no consistent specificity relative to other LR tracts. These results suggest that directional coherence and dispersion may be shaped by more global white matter organization, or may be less sensitive to localized structural associations. Together, these findings indicate that SAF‐LR coupling is robust for metrics sensitive to tissue composition and volume fractions, but less so for those emphasizing geometric complexity, reflecting potentially distinct biological constraints across microstructural domains.

### 4.5 Limitations and Future Directions

There are several limitations that are important to acknowledge. While this study leveraged a high‐resolution dataset that reduces variability in quantification, diffusion tractography is influenced by partial volume effects (Schilling, Archer, Yeh, et al. 2023). In addition, smaller brain sizes in development may be subject to greater partial volume effects as one voxel takes up a larger relative proportion of the brain. The relationships between features (i.e., paired LR‐SAFs) are also confounded by partial volume effects, possibly inflating feature‐wise or pathway‐based cross‐sectional correlations. A valuable avenue for future research is the development and application of more sophisticated modeling frameworks to address challenges with covariance among imaging metrics and partial volume effects and to study longitudinal changes with age. Furthermore, while these trends across age and sex are statistically significant, the effect sizes are modest, limiting their applicability to subject‐specific inference or classification using a single metric alone. Nevertheless, the findings in this study inform our understanding of normative trajectories across development, which will be useful for comparisons to investigate atypical development and dysfunction in disease pathology.

## 5 Conclusion

This study applied advanced quantitative diffusion tractography methods to map SAFs in relation to known long‐range white matter tracts in a large‐scale developmental cohort over the ages of 5.6–21.9 years old. Uniting advanced DTI and NODDI diffusion models, we comprehensively characterized the associations of SAF and long‐range bundle microstructure with age and sex as well as the relationships between microstructural features in corresponding SAF and long‐range bundles. In addition to significant changes in microstructure over developmental age across all tracts, significant differences were identified between SAF and long‐range bundles, suggesting distinct neurodevelopmental trajectories between superficial and deep white matter. Furthermore, we identified significant sex and age‐sex interaction effects on white matter development, suggesting differing developmental trajectories between males and females. This normative study provides insights into typical changes in neuronal structure, organization, and integrity in SAFs and associated long‐range white matter tracts during the important period from childhood to young adulthood, laying a foundation for future research to investigate atypical changes and dysfunction in disease pathology.

## Author Contributions

C.C.:original draft, conceptualization, methodology, analysis, visualizations, reviewing, editing. M.C.: conceptualization, methodology, reviewing, editing. F.R.: methodology, reviewing, editing. D.M.: methodology, reviewing, editing. B.A.L.: funding, conceptualization, methodology, analysis, reviewing, editing, supervision. K.G.S.: funding, conceptualization, methodology, analysis, reviewing, editing, supervision.

## Conflicts of Interest

The authors declare no conflicts of interest.

## Supporting information


**Data S1.** Supporting Information.

## Data Availability

The data that support the findings of this study are openly available in Human Connectome Project Development (HCP‐D) at https://nda.nih.gov/study.html?id=1063.
