# Supplementary material for: Microstructural Characterization of Short Association Fibers Related to Long‐Range White Matter Tracts in Normative Development
Source: Hum Brain Mapp. 2025 Jun 9;46(8):e70255. doi: 10.1002/hbm.70255 (PMC12148645; doi:10.1002/hbm.70255)
Supplement: Supplementary file 1 — Data S1. Supporting Information. [file HBM-46-e70255-s001.docx]

**Microstructural Characterization of Short Association Fibers Related to Long-Range White Matter Tracts in Normative Development**

Chloe Cho^a,*^, Maxime Chamberland^b^, Francois Rheault^c^, Daniel Moyer^d^, Bennett A. Landman^a,d,e,f,g^, Kurt G. Schilling^e,g^

^a^Department of Biomedical Engineering, Vanderbilt University, Nashville, TN, USA

^b^Department of Mathematics and Computer Science, Eindhoven University of Technology, Eindhoven, The Netherlands

^c^Medical Imaging and Neuroinformatic (MINi) Lab, Department of Computer Science, University of Sherbrooke, Canada

^d^Department of Computer Science, Vanderbilt University, Nashville, TN, USA

^e^Department of Radiology and Radiological Sciences, Vanderbilt University, Nashville, TN, USA

^f^Department of Electrical and Computer Engineering, Vanderbilt University, Nashville, TN, USA

^g^Vanderbilt University Institute of Imaging Science, Vanderbilt University, Nashville, TN, USA

*Corresponding Author: Chloe Cho (chloe.cho@vanderbilt.edu)

**Supplementary Information**

“Microstructural Characterization of Short Association Fibers Related to Long-Range White Matter Tracts in Normative Development”

**Supplementary Table 1**. TractSeg-Based Classification of White Matter Tracts

| **Association Pathways** | |
| --- | --- |
| AF_left | Arcuate Fascicle Left |
| AF_right | Arcuate Fascicle Right |
| CG_left | Cingulum Left |
| CG_right | Cingulum Right |
| IFO_left | Inferior Occipito-Frontal Fascicle Left |
| IFO_right | Inferior Occipito-Frontal Fascicle Right |
| ILF_left | Inferior Longitudinal Fascicle Left |
| ILF_right | Inferior Longitudinal Fascicle Right |
| MLF_left | Middle Longitudinal Fascicle Left |
| MLF_right | Middle Longitudinal Fascicle Right |
| SLF_I_left | Superior Longitudinal Fascicle I Left |
| SLF_I_right | Superior Longitudinal Fascicle I Right |
| SLF_II_left | Superior Longitudinal Fascicle II Left |
| SLF_II_right | Superior Longitudinal Fascicle II Right |
| SLF_III_left | Superior Longitudinal Fascicle III Left |
| SLF_III_right | Superior Longitudinal Fascicle III Right |
| UF_left | Uncinate Fascicle Left |
| UF_right | Uncinate Fascicle Right |
| **Commissural Pathways** | |
| CC_1 | Rostrum |
| CC_2 | Genu |
| CC_3 | Rostral Body (Premotor) |
| CC_4 | Anterior Midbody (Primary Motor) |
| CC_5 | Posterior Midbody (Primary Somatosensory) |
| CC_6 | Isthmus |
| CC_7 | Splenium |
| **Projection Pathways** | |
| ATR_left | Anterior Thalamic Radiation Left |
| ATR_right | Anterior Thalamic Radiation Right |
| CST_left | Corticospinal Tract Left |
| CST_right | Corticospinal Tract Right |
| FPT_left | Fronto-Pontine Tract Left |
| FPT_right | Fronto-Pontine Tract Right |
| OR_left | Optic Radiation Left |
| OR_right | Optic Radiation Right |
| POPT_left | Parieto‐Occipital Pontine Left |
| POPT_right | Parieto‐Occipital Pontine Right |
| STR_left | Superior Thalamic Radiation Left |
| STR_right | Superior Thalamic Radiation Right |
| **Striatal Pathways** | |
| ST_FO_left | Striato-Fronto-Orbital Left |
| ST_FO_right | Striato-Fronto-Orbital Right |
| ST_OCC_left | Striato-Occipital Left |
| ST_OCC_right | Striato-Occipital Right |
| ST_PAR_left | Striato-Parietal Left |
| ST_PAR_right | Striato-Parietal Right |
| ST_POSTC_left | Striato-Postcentral Left |
| ST_POSTC_right | Striato-Postcentral Right |
| ST_PREC_left | Striato-Precentral Left |
| ST_PREC_right | Striato-Precentral Right |
| ST_PREF_left | Striato-Prefrontal Left |
| ST_PREF_right | Striato-Prefrontal Right |
| ST_PREM_left | Striato-Premotor Left |
| ST_PREM_right | Striato-Premotor Right |
| **Thalamic Pathways** | |
| T_OCC_left | Thalamo-Occipital Left |
| T_OCC_right | Thalamo-Occipital Right |
| T_PAR_left | Thalamo-Parietal Left |
| T_PAR_right | Thalamo-Parietal Right |
| T_POSTC_left | Thalamo-Postcentral Left |
| T_POSTC_right | Thalamo-Postcentral Right |
| T_PREC_left | Thalamo-Precentral Left |
| T_PREC_right | Thalamo-Precentral Right |
| T_PREF_left | Thalamo-Prefrontal Left |
| T_PREF_right | Thalamo-Prefrontal Right |
| T_PREM_left | Thalamo-Premotor Left |
| T_PREM_right | Thalamo-Premotor Right |

**Supplementary Table 2**. Quantitative Data Table with Cross-Sectional Relative Change in Feature Per Year as a Percent of the Population Mean for All Features and Regions of Interest

| **SAF Bundle** | **FA** | **MD** | **AD** | **RD** | **ICVF** | **ISOVF** | **ODI** |
| --- | --- | --- | --- | --- | --- | --- | --- |
| AF_left | 0.19 | -0.52 | -0.47 | -0.58 | 1.40 | 4.39 | 0.67 |
| AF_right | 0.21 | -0.51 | -0.45 | -0.58 | 1.43 | 4.76 | 0.65 |
| CG_left | 0.27 | -0.52 | -0.42 | -0.62 | 1.38 | 3.63 | 0.54 |
| CG_right | 0.21 | -0.51 | -0.44 | -0.59 | 1.31 | 3.51 | 0.54 |
| IFO_left | 0.15 | -0.46 | -0.43 | -0.49 | 1.30 | 3.89 | 0.63 |
| IFO_right | 0.14 | -0.45 | -0.41 | -0.49 | 1.29 | 4.00 | 0.63 |
| ILF_left | 0.17 | -0.45 | -0.41 | -0.48 | 1.26 | 3.30 | 0.61 |
| ILF_right | 0.17 | -0.43 | -0.39 | -0.47 | 1.29 | 3.88 | 0.61 |
| MLF_left | 0.24 | -0.51 | -0.44 | -0.58 | 1.37 | 4.46 | 0.62 |
| MLF_right | 0.24 | -0.50 | -0.43 | -0.56 | 1.37 | 4.53 | 0.61 |
| SLF_I_left | 0.37 | -0.59 | -0.46 | -0.72 | 1.46 | 4.27 | 0.51 |
| SLF_I_right | 0.35 | -0.56 | -0.45 | -0.68 | 1.42 | 4.28 | 0.50 |
| SLF_II_left | 0.24 | -0.56 | -0.49 | -0.65 | 1.40 | 4.07 | 0.62 |
| SLF_II_right | 0.24 | -0.55 | -0.47 | -0.64 | 1.39 | 4.20 | 0.62 |
| SLF_III_left | 0.18 | -0.53 | -0.47 | -0.59 | 1.37 | 4.34 | 0.67 |
| SLF_III_right | 0.19 | -0.51 | -0.45 | -0.57 | 1.37 | 4.75 | 0.64 |
| UF_left | 0.01 | -0.44 | -0.44 | -0.45 | 1.35 | 5.14 | 0.80 |
| UF_right | 0.01 | -0.45 | -0.44 | -0.47 | 1.38 | 4.75 | 0.82 |
| CC_1 | 0.04 | -0.46 | -0.45 | -0.47 | 1.49 | 4.81 | 0.81 |
| CC_2 | 0.25 | -0.54 | -0.46 | -0.63 | 1.44 | 3.93 | 0.58 |
| CC_3 | 0.29 | -0.58 | -0.47 | -0.70 | 1.39 | 3.31 | 0.50 |
| CC_4 | 0.28 | -0.62 | -0.52 | -0.71 | 1.43 | 3.93 | 0.69 |
| CC_5 | 0.25 | -0.55 | -0.48 | -0.63 | 1.35 | 4.10 | 0.68 |
| CC_6 | 0.24 | -0.50 | -0.42 | -0.57 | 1.30 | 3.35 | 0.59 |
| CC_7 | 0.21 | -0.43 | -0.39 | -0.48 | 1.18 | 2.42 | 0.55 |
| ATR_left | 0.10 | -0.51 | -0.47 | -0.55 | 1.38 | 4.51 | 0.71 |
| ATR_right | 0.10 | -0.52 | -0.48 | -0.56 | 1.38 | 4.46 | 0.69 |
| CST_left | 0.27 | -0.64 | -0.55 | -0.73 | 1.50 | 4.79 | 0.73 |
| CST_right | 0.33 | -0.63 | -0.52 | -0.74 | 1.54 | 5.41 | 0.69 |
| FPT_left | 0.23 | -0.56 | -0.47 | -0.65 | 1.38 | 3.99 | 0.62 |
| FPT_right | 0.27 | -0.55 | -0.46 | -0.65 | 1.39 | 4.13 | 0.54 |
| OR_left | 0.11 | -0.45 | -0.45 | -0.46 | 1.18 | 2.73 | 0.66 |
| OR_right | 0.14 | -0.43 | -0.41 | -0.46 | 1.16 | 3.02 | 0.63 |
| POPT_left | 0.24 | -0.52 | -0.45 | -0.59 | 1.31 | 4.48 | 0.65 |
| POPT_right | 0.25 | -0.52 | -0.45 | -0.59 | 1.31 | 4.26 | 0.62 |
| STR_left | 0.24 | -0.60 | -0.52 | -0.67 | 1.37 | 4.90 | 0.76 |
| STR_right | 0.21 | -0.58 | -0.51 | -0.64 | 1.34 | 4.78 | 0.75 |
| ST_FO_left | -0.02 | -0.46 | -0.47 | -0.46 | 1.41 | 5.15 | 0.87 |
| ST_FO_right | 0.00 | -0.47 | -0.47 | -0.48 | 1.44 | 5.01 | 0.85 |
| ST_OCC_left | 0.11 | -0.45 | -0.43 | -0.47 | 1.19 | 2.87 | 0.65 |
| ST_OCC_right | 0.17 | -0.43 | -0.39 | -0.47 | 1.21 | 3.48 | 0.59 |
| ST_PAR_left | 0.23 | -0.51 | -0.44 | -0.58 | 1.32 | 4.30 | 0.63 |
| ST_PAR_right | 0.24 | -0.50 | -0.43 | -0.57 | 1.31 | 4.04 | 0.60 |
| ST_POSTC_left | 0.21 | -0.53 | -0.48 | -0.59 | 1.36 | 5.17 | 0.73 |
| ST_POSTC_right | 0.16 | -0.52 | -0.47 | -0.56 | 1.34 | 5.26 | 0.77 |
| ST_PREC_left | 0.26 | -0.58 | -0.50 | -0.66 | 1.44 | 4.70 | 0.67 |
| ST_PREC_right | 0.28 | -0.56 | -0.47 | -0.65 | 1.45 | 5.16 | 0.65 |
| ST_PREF_left | 0.23 | -0.53 | -0.46 | -0.61 | 1.45 | 4.48 | 0.62 |
| ST_PREF_right | 0.23 | -0.53 | -0.45 | -0.61 | 1.44 | 4.49 | 0.59 |
| ST_PREM_left | 0.31 | -0.57 | -0.46 | -0.68 | 1.45 | 3.85 | 0.47 |
| ST_PREM_right | 0.31 | -0.56 | -0.44 | -0.68 | 1.44 | 4.18 | 0.47 |
| T_OCC_left | 0.15 | -0.45 | -0.43 | -0.47 | 1.19 | 2.77 | 0.62 |
| T_OCC_right | 0.17 | -0.43 | -0.40 | -0.46 | 1.17 | 3.23 | 0.60 |
| T_PAR_left | 0.27 | -0.51 | -0.44 | -0.59 | 1.34 | 4.44 | 0.62 |
| T_PAR_right | 0.32 | -0.51 | -0.42 | -0.60 | 1.35 | 4.42 | 0.54 |
| T_POSTC_left | 0.27 | -0.55 | -0.48 | -0.62 | 1.40 | 5.47 | 0.69 |
| T_POSTC_right | 0.24 | -0.54 | -0.47 | -0.60 | 1.39 | 5.46 | 0.70 |
| T_PREC_left | 0.33 | -0.62 | -0.51 | -0.72 | 1.51 | 4.88 | 0.63 |
| T_PREC_right | 0.34 | -0.61 | -0.49 | -0.72 | 1.52 | 5.30 | 0.62 |
| T_PREF_left | 0.21 | -0.54 | -0.47 | -0.61 | 1.42 | 4.29 | 0.65 |
| T_PREF_right | 0.22 | -0.54 | -0.46 | -0.62 | 1.43 | 4.32 | 0.61 |
| T_PREM_left | 0.35 | -0.60 | -0.47 | -0.73 | 1.50 | 3.77 | 0.43 |
| T_PREM_right | 0.34 | -0.59 | -0.46 | -0.72 | 1.48 | 4.12 | 0.46 |
| **LR Bundle** | **FA** | **MD** | **AD** | **RD** | **ICVF** | **ISOVF** | **ODI** |
| AF_left | 0.55 | -0.56 | -0.38 | -0.74 | 1.55 | 3.91 | 0.17 |
| AF_right | 0.55 | -0.55 | -0.36 | -0.74 | 1.54 | 4.03 | 0.10 |
| CG_left | 0.68 | -0.51 | -0.27 | -0.76 | 1.56 | 4.27 | 0.03 |
| CG_right | 0.67 | -0.51 | -0.28 | -0.75 | 1.55 | 4.41 | -0.05 |
| IFO_left | 0.43 | -0.36 | -0.22 | -0.50 | 1.34 | 3.44 | 0.21 |
| IFO_right | 0.46 | -0.35 | -0.20 | -0.50 | 1.35 | 3.52 | 0.17 |
| ILF_left | 0.62 | -0.41 | -0.22 | -0.59 | 1.41 | 3.91 | 0.01 |
| ILF_right | 0.55 | -0.41 | -0.23 | -0.58 | 1.42 | 4.13 | 0.13 |
| MLF_left | 0.58 | -0.48 | -0.28 | -0.68 | 1.51 | 4.25 | 0.16 |
| MLF_right | 0.59 | -0.48 | -0.27 | -0.68 | 1.52 | 4.43 | 0.10 |
| SLF_I_left | 0.60 | -0.57 | -0.36 | -0.79 | 1.58 | 4.51 | 0.02 |
| SLF_I_right | 0.65 | -0.55 | -0.32 | -0.80 | 1.55 | 4.25 | -0.10 |
| SLF_II_left | 0.49 | -0.55 | -0.39 | -0.72 | 1.50 | 3.54 | 0.18 |
| SLF_II_right | 0.55 | -0.55 | -0.35 | -0.75 | 1.52 | 3.74 | 0.11 |
| SLF_III_left | 0.55 | -0.54 | -0.35 | -0.72 | 1.51 | 3.68 | 0.13 |
| SLF_III_right | 0.53 | -0.52 | -0.33 | -0.68 | 1.51 | 4.05 | 0.15 |
| UF_left | 0.27 | -0.45 | -0.37 | -0.53 | 1.44 | 5.22 | 0.41 |
| UF_right | 0.22 | -0.43 | -0.37 | -0.51 | 1.44 | 5.36 | 0.53 |
| CC_1 | 0.28 | -0.39 | -0.29 | -0.50 | 1.46 | 3.67 | 0.48 |
| CC_2 | 0.45 | -0.47 | -0.30 | -0.66 | 1.38 | 2.85 | 0.12 |
| CC_3 | 0.52 | -0.52 | -0.32 | -0.72 | 1.45 | 2.53 | 0.03 |
| CC_4 | 0.48 | -0.56 | -0.37 | -0.75 | 1.42 | 2.57 | 0.27 |
| CC_5 | 0.41 | -0.41 | -0.30 | -0.55 | 1.20 | 2.31 | 0.18 |
| CC_6 | 0.57 | -0.43 | -0.21 | -0.68 | 1.35 | 2.64 | -0.04 |
| CC_7 | 0.54 | -0.22 | -0.04 | -0.44 | 1.09 | 2.13 | -0.19 |
| ATR_left | 0.50 | -0.46 | -0.30 | -0.61 | 1.42 | 3.83 | 0.11 |
| ATR_right | 0.48 | -0.47 | -0.31 | -0.62 | 1.43 | 3.88 | 0.13 |
| CST_left | 0.28 | -0.48 | -0.37 | -0.62 | 1.16 | 2.20 | 0.43 |
| CST_right | 0.33 | -0.48 | -0.34 | -0.64 | 1.17 | 2.52 | 0.35 |
| FPT_left | 0.40 | -0.47 | -0.32 | -0.65 | 1.23 | 2.47 | 0.15 |
| FPT_right | 0.42 | -0.46 | -0.30 | -0.65 | 1.22 | 2.52 | 0.11 |
| OR_left | 0.53 | -0.32 | -0.16 | -0.49 | 1.21 | 2.90 | 0.04 |
| OR_right | 0.51 | -0.30 | -0.13 | -0.47 | 1.16 | 2.96 | -0.01 |
| POPT_left | 0.46 | -0.40 | -0.24 | -0.59 | 1.22 | 2.77 | 0.00 |
| POPT_right | 0.46 | -0.40 | -0.22 | -0.59 | 1.17 | 2.67 | 0.00 |
| STR_left | 0.24 | -0.43 | -0.36 | -0.51 | 1.09 | 3.66 | 0.26 |
| STR_right | 0.29 | -0.42 | -0.32 | -0.52 | 1.10 | 3.90 | 0.24 |
| ST_FO_left | 0.38 | -0.44 | -0.32 | -0.55 | 1.48 | 4.12 | 0.31 |
| ST_FO_right | 0.28 | -0.43 | -0.36 | -0.52 | 1.47 | 4.24 | 0.44 |
| ST_OCC_left | 0.52 | -0.30 | -0.14 | -0.48 | 1.21 | 3.14 | -0.01 |
| ST_OCC_right | 0.57 | -0.29 | -0.10 | -0.48 | 1.23 | 3.27 | -0.07 |
| ST_PAR_left | 0.54 | -0.44 | -0.24 | -0.65 | 1.37 | 3.80 | -0.02 |
| ST_PAR_right | 0.57 | -0.43 | -0.21 | -0.65 | 1.37 | 3.93 | -0.11 |
| ST_POSTC_left | 0.51 | -0.41 | -0.25 | -0.57 | 1.31 | 3.97 | 0.01 |
| ST_POSTC_right | 0.57 | -0.40 | -0.21 | -0.59 | 1.30 | 4.28 | -0.09 |
| ST_PREC_left | 0.51 | -0.52 | -0.34 | -0.70 | 1.42 | 3.63 | 0.09 |
| ST_PREC_right | 0.50 | -0.51 | -0.33 | -0.70 | 1.40 | 3.97 | 0.12 |
| ST_PREF_left | 0.52 | -0.50 | -0.33 | -0.67 | 1.48 | 3.86 | 0.10 |
| ST_PREF_right | 0.50 | -0.50 | -0.33 | -0.67 | 1.47 | 3.89 | 0.11 |
| ST_PREM_left | 0.56 | -0.53 | -0.34 | -0.72 | 1.41 | 3.02 | -0.03 |
| ST_PREM_right | 0.59 | -0.53 | -0.31 | -0.75 | 1.43 | 3.42 | -0.11 |
| T_OCC_left | 0.51 | -0.30 | -0.15 | -0.46 | 1.21 | 3.00 | 0.05 |
| T_OCC_right | 0.54 | -0.29 | -0.12 | -0.46 | 1.17 | 3.02 | -0.02 |
| T_PAR_left | 0.52 | -0.43 | -0.25 | -0.62 | 1.33 | 3.87 | 0.03 |
| T_PAR_right | 0.54 | -0.41 | -0.22 | -0.61 | 1.31 | 3.91 | 0.00 |
| T_POSTC_left | 0.46 | -0.41 | -0.27 | -0.55 | 1.22 | 3.80 | 0.08 |
| T_POSTC_right | 0.49 | -0.39 | -0.22 | -0.56 | 1.19 | 4.00 | 0.01 |
| T_PREC_left | 0.41 | -0.52 | -0.38 | -0.67 | 1.31 | 3.38 | 0.26 |
| T_PREC_right | 0.43 | -0.52 | -0.36 | -0.68 | 1.33 | 3.73 | 0.24 |
| T_PREF_left | 0.51 | -0.50 | -0.32 | -0.69 | 1.40 | 3.71 | 0.04 |
| T_PREF_right | 0.52 | -0.50 | -0.32 | -0.68 | 1.40 | 3.74 | 0.02 |
| T_PREM_left | 0.55 | -0.51 | -0.31 | -0.70 | 1.33 | 3.05 | -0.07 |
| T_PREM_right | 0.58 | -0.51 | -0.29 | -0.72 | 1.36 | 3.31 | -0.10 |


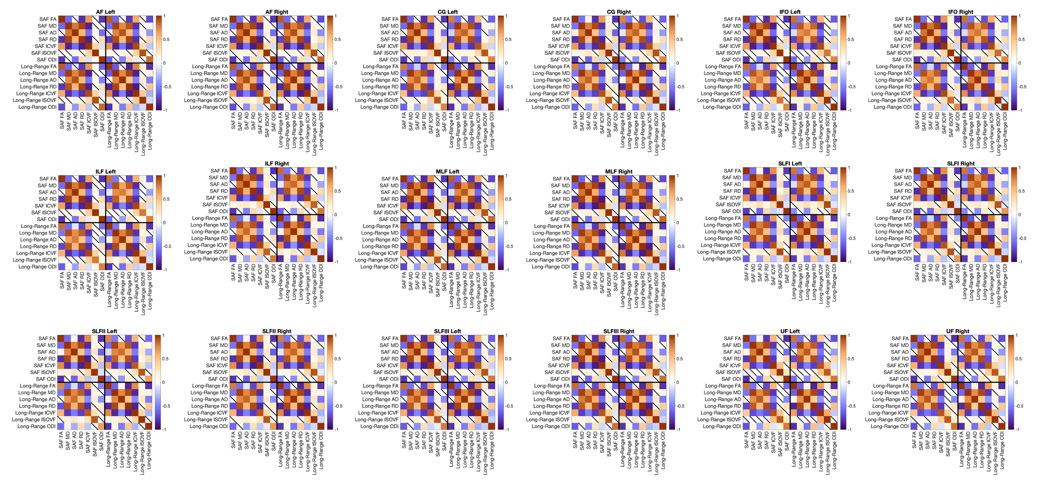


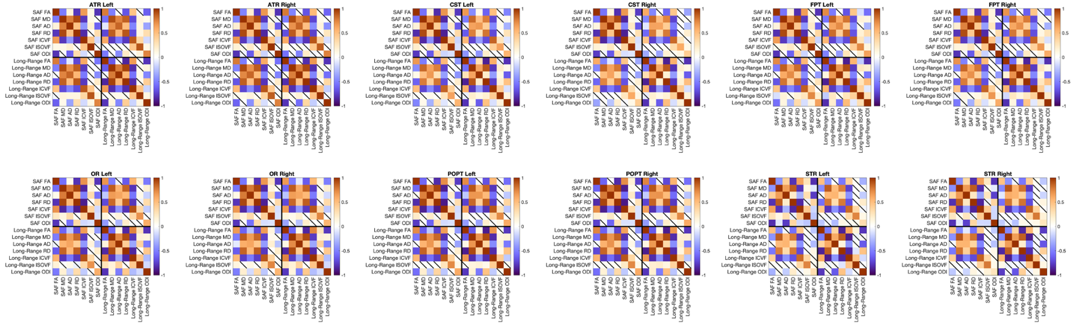


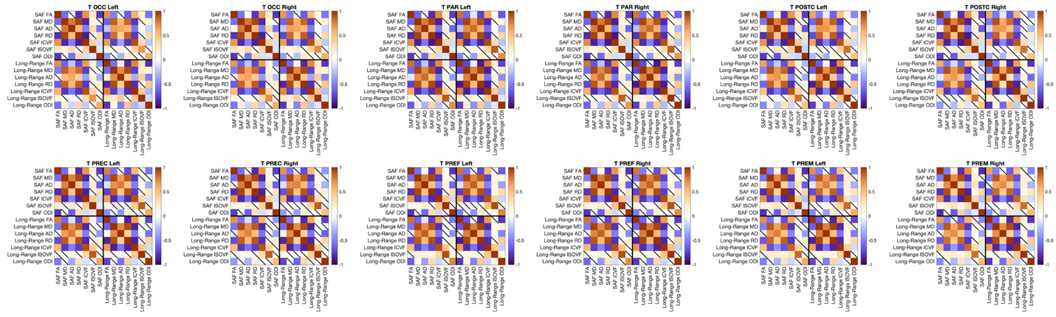


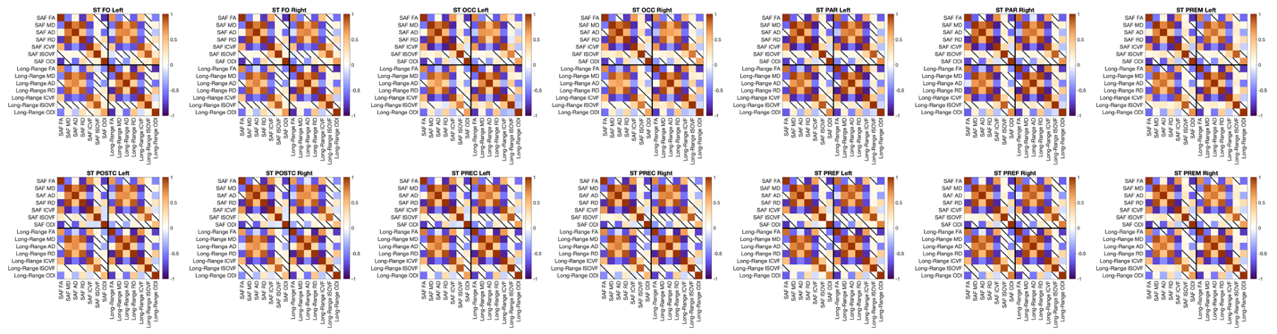


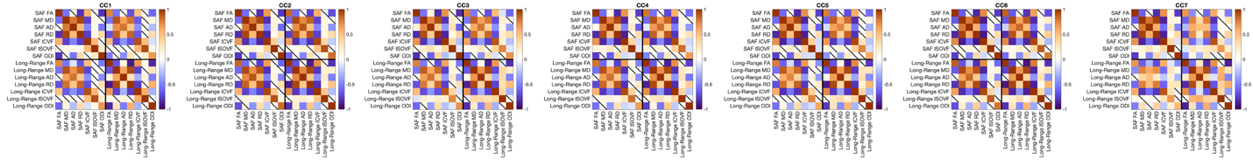


**Supplementary Fig. 1**. Long-range and SAF bundles demonstrate similar overall correlational patterns when assessing (1) the relationship of features within each SAF bundle, (2) the relationship of features within each long-range bundle, and (3) the relationship of SAF bundle features with its corresponding long-range bundle features. There is variation in the strength and significance of feature correlations among different pathways. For all pathway classifications, partial correlation coefficients are shown with significance determined by alpha=0.05 after FDR correction (non-significant correlations are shown with a diagonal line).
